# Profiling of microRNAs in tumor interstitial fluid of breast tumors – a novel resource to identify biomarkers for prognostic classification and detection of cancer

**DOI:** 10.1002/1878-0261.12025

**Published:** 2016-12-12

**Authors:** Ann Rita Halvorsen, Åslaug Helland, Pavel Gromov, Vera Timmermans Wielenga, Maj‐Lis Møller Talman, Nils Brunner, Vandana Sandhu, Anne‐Lise Børresen‐Dale, Irina Gromova, Vilde D. Haakensen

**Affiliations:** ^1^ Department of Cancer Genetics Institute for Cancer Research The Norwegian Radium Hospital Oslo University Hospital Norway; ^2^ Department of Oncology The Norwegian Radium Hospital Oslo University Hospital Norway; ^3^ Danish Cancer Society Research Center Genome Integrity Unit Cancer Proteomics Group Copenhagen Denmark; ^4^ Department of Pathology Center of Diagnostic Investigations Copenhagen University Hospital Denmark; ^5^ Section for Molecular Disease Biology and Sino‐Danish Breast Cancer Research Center Department of Veterinary Disease Biology Faculty of Health and Medical Sciences University of Copenhagen Denmark; ^6^ Translational Cancer Research Unit Danish Cancer Society Copenhagen Denmark; ^7^ Institute for Clinical Medicine Faculty of Medicine University of Oslo Norway

**Keywords:** biomarker, breast cancer, cross‐talk, microenvironment, microRNA, tumor interstitial fluid

## Abstract

It has been hypothesized based on accumulated data that a class of small noncoding RNAs, termed microRNAs, are key factors in intercellular communication. Here, microRNAs present in interstitial breast tumor fluids have been analyzed to identify relevant markers for a diagnosis of breast cancer and to elucidate the cross‐talk that exists among cells in a tumor microenvironment. Matched tumor interstitial fluid samples (TIF,* n* = 60), normal interstitial fluid samples (NIF,* n* = 51), corresponding tumor tissue specimens (*n* = 54), and serum samples (*n* = 27) were collected from patients with breast cancer, and detectable microRNAs were analyzed and compared. In addition, serum data from 32 patients with breast cancer and 22 healthy controls were obtained for a validation study. To identify potential serum biomarkers of breast cancer, first the microRNA profiles of TIF and NIF samples were compared. A total of 266 microRNAs were present at higher level in the TIF samples as compared to normal counterparts. Sixty‐one of these microRNAs were present in > 75% of the serum samples and were subsequently tested in a validation set. Seven of the 61 microRNAs were associated with poor survival, while 23 were associated with the presence of immune cells and adipocytes. To our knowledge, these data demonstrate for the first time that profiling of microRNAs in TIF can identify novel biomarkers for the prognostic classification and detection of breast cancer. In addition, the present findings demonstrate that microRNAs may represent the cross‐talk that occurs between tumor cells and their surrounding stroma.

**Abbreviations**
DFS: disease‐free survival
ER: estrogen receptor
FDR: false discovery rate
FFPE: formalin‐fixed paraffin‐embedded
HER2: human epidermal factor 2
IHC: immunohistochemistry
NIF: normal interstitial fluid
PgR: progesterone receptor
TAMs: tumor‐associated macrophages
TIF: tumor interstitial fluid
TILs: tumor‐infiltrating lymphocytes
TLDA: TaqMan® Low Density Arrays
TNBC: triple‐negative breast tumors

## 1 Introduction

Breast tumor heterogeneity in terms of morphology and clinical outcome is well known, and these observations have mainly been based on pathology‐driven classifications of breast tumors. However, despite a refinement of morphological subtyping of breast tumors more than a decade ago, molecular classifications of breast tumors based on gene expression and/or protein marker profiling are currently employed (Perou *et al*., 2000; Prat and Perou, 2011). Consistent with the heterogeneous pathology observed for breast cancers on both molecular and cellular levels, recent studies have revealed extensive genetic diversity within the same breast cancer subtype, and each breast tumor appears to have a unique molecular composition. Such intertumor, or clonal, heterogeneity affects key cancer pathways and drives phenotypic variations (Kao *et al*., 2009; Mackay *et al*., 2009) while also presenting a significant challenge in the treatment for breast cancer.

Breast cancer tumors are composed of a number of different cells, including malignant cells, stromal components, infiltrating host cells, and adjacent normal tissue cells (de Visser *et al*., 2006). As such, the heterogeneity in the tumor microenvironment, intratumor heterogeneity, represents a major challenge to efforts intended to identify tailored biomarkers for use in clinical practice. Ideally, a blood‐based test for breast cancer detection would facilitate both diagnostic and follow‐up settings for clinicians as a supplement to mammography. Thus, significant effort has been invested to identify noninvasive biomarkers for the diagnosis, prediction of therapy response, and prognosis of breast cancer (Bertoli *et al*., 2015).

A novel approach for breast cancer biomarker research has recently been developed at the Danish Cancer Society Research Center, and it involves the collection and analysis of breast tumor interstitial fluid (TIF; Gromov *et al*., 2013). Compounds detected in interstitial fluid samples derive from all of the cells present within a tumor mass, and contain signaling molecules that underlie the interplay between tumor cells and their microenvironment. It is hypothesized that biomolecules such as microRNAs, lipids, and proteins that are secreted by a tumor and stromal cells into the interstitium are drained by the lymphatic system and enter the bloodstream (Wagner and Wiig, 2015). Thus, these biomolecules could potentially be detected and quantified. However, to date, no robust blood markers for breast cancer have been identified.

It has been demonstrated and confirmed that the spatial organization of multiple cell types within tumors, including adipose cells, tumor‐associated macrophages (TAMs), and multiple tumor‐infiltrating lymphocytes (TILs), plays an essential role in tumor development and drug responsiveness (Swartz *et al*., 2012). Moreover, this organization is often reported to impact prognosis (Salgado *et al*., 2015). For example, high levels of TAMs are often associated with poor prognosis in breast cancer (Ruffell *et al*., 2012). Regarding TILs, a comprehensive meta‐analysis found that TILs only moderately affect cancer prognosis, while the ratio of lymphocyte subgroups could potentially be more informative (Gooden *et al*., 2011). Correspondingly, different subtypes of leukocytes, such as CD8^+^ cytotoxic T lymphocytes and CD4^+^ T‐helper cells, are often associated with good prognosis (Ruffell *et al*., 2012), while anti‐CD4^+^ cells, which can be a marker for T regulatory cells, have been associated with a less favorable prognosis in some cases (Li *et al*., 2014).

microRNAs constitute a distinct class of small noncoding RNAs that are recognized as gene expression modulators. microRNAs have also been found to negatively regulate numerous mRNAs by silencing their target transcript (Ma and Weinberg, 2008). Accumulating evidence indicates that the expression of microRNAs in cancer and stromal cells during tumor progression is altered (Soon and Kiaris, 2013). Furthermore, several microRNAs are recognized to be crucial regulators of immune cells. However, it remains unclear whether microRNAs are pivotal to the communication between tumor‐associated immune cells and tumor cells (Frediani and Fabbri, 2016; Kohlhapp *et al*., 2015).

Recently, microRNAs have been examined in various body fluids, including blood, saliva, urine, cerebrospinal fluid, and breast milk. Each type of body fluid exhibited a distinct microRNA pattern, which potentially represents a tissue‐specific profile (Weber *et al*., 2010). Circulating microRNAs have been found to be very stable, and tumor cells release a greater number of microRNAs than normal cells (Riches *et al*., 2014). Distinct alterations in microRNA expression profiles have also been observed during cancer progression (Tahiri *et al*., 2014), thereby identifying microRNAs as attractive molecules to serve as biomarkers.

Until now, analyses of the molecular components of TIF samples have been protein based (Celis *et al*., 2004). In the present study, microRNAs in TIF released from tumors were compared with microRNAs released from normal mammary tissue (NIF) and matched serum samples. The aim of the present study was to detect microRNAs that are secreted from tumor and stromal cells in order to identify possible serum‐based microRNA biomarkers for breast cancer. In addition, a correlation between elevated levels of microRNAs in TIF samples and the presence of TILs and adipocytes was observed. The present study confirms that breast tumor‐associated microRNA‐based signatures are detectable in blood. Furthermore, the role of microRNAs as mediators in the complex interplay between a tumor and the stromal environment has been elucidated.

## 2 Materials and methods

### 2.1 Sample collection and handling

Tissue samples, including the tumor and corresponding normal tissue distant from the tumor margin, were collected by the Danish Center for Translational Breast Cancer Research Program from clinical high‐risk patients (for criteria, see Doc S1) who underwent a mastectomy between 2003 and 2012. None of the patients had previously undergone surgery involving the breast and they did not receive preoperative treatment. The patients presented a unifocal tumor with an estimated size > 20 mm. Patient survival, subsequently referred to as disease‐free survival (DFS), was measured from the time of surgery until the date of first recurrence or the date of death from breast cancer. If the patient remained alive, or died due to other causes, the patient was censored. Death records up to December 31, 2013, were included, and these were confirmed in the Danish Cancer Registration System and the Danish Register of Causes of Death. Clinicopathological information was provided by the Department of Pathology, Rigshospitalet, Copenhagen University Hospital. This study was conducted in compliance with the Helsinki II Declaration, and written informed consent was obtained from all participants. This project was approved by the Copenhagen and Frederiksberg regional division of the Danish National Committee on Biomedical Research Ethics (KF 01‐069/03).

Each tissue specimen was divided into two pieces. One tissue piece was stored at −80 °C and was later used for microRNA extraction and the preparation of formalin‐fixed and paraffin‐embedded (FFPE) blocks for histological characterization, tumor subtyping, and scoring of TILs. The second tissue piece was used for interstitial fluid recovery (TIF and NIF) and was stored in PBS buffer at 4 °C within a maximum of 45 min from the time of surgical excision. Blood samples were collected preoperatively according to a standardized protocol (Wurtz *et al*., 2008).

### 2.2 Sample characterization, breast tumor subtyping, and scoring of TILs

Immunohistochemistry (IHC) was performed as previously described to fulfill histological characterizations (Gromov *et al*., 2010). The first and last sections of each FFPE sample were stained with a CK19 antibody to verify tumor cell content and the number of mammary epithelial glands (Gromov *et al*., 2010). A visual assessment of tumor stroma percentage, as well as the proportion of adipose tissue, was evaluated as previously described (Mesker *et al*., 2007). Slides were reviewed by two blinded researchers (IIG and PSG). Subtype scoring of luminal A, luminal B, HER2, and triple‐negative tumors (TNBC) was performed based on estrogen receptor (ER), progesterone receptor (PgR), human epidermal receptor‐2 (HER2), and Ki67 status in accordance with St. Gallen International Breast Cancer Guidelines (Esposito *et al*., 2015) (Table S1). An overview of the samples examined and the tumor subtypes identified are listed in Tables 1 and S2. The antibodies used for ER, PgR, HER2, and Ki67 scoring are listed in Table S3. The proportion of TILs in direct contact with the tumors was evaluated by IHC in accordance with the recommendations of the International TILs Working Group 2014 (Salgado *et al*., 2015). Scoring of total TILs (1+ to 3+) was performed based on an evaluation of the sections stained by hematoxylin and eosin (HE) with the following categories: 1+ – ≤ 10%; 2+ – 10–50%; and 3+ – > 50% of the cells stained. Scoring of total leukocytes, T lymphocytes, T‐helper lymphocytes, cytotoxic T lymphocytes, and macrophages was determined based on staining performed with antibodies raised against CD45^+^, CD3^+^, CD4^+^, CD8^+^, and CD68^+^, respectively. In Fig. S1, an example of the leukocyte evaluation that was performed with the most common CD markers is shown. Only antibodies whose specificity was previously evaluated in a number of published studies were used (see Table S3).

**Table 1:** Stratification of samples analyzed in this study according to tumor subtype, grade, and ER, PgR, and HER2 status. Scoring of the clinical features for the TIF, NIF, and serum samples refers to the corresponding tumor material. NIF, normal interstitial fluid; TIF, tumor interstitial fluid; ER, estrogen receptor; PgR, progesterone receptor; HER2, human epidermal factor 2

	Tumor	TIF	NIF	Serum	*P* value^a^
Number of cases	54	60	51	27	
Subtype					
Lum A	24	25	23	12	0.98
Lum B	12	14	13	6	
HER2	7	8	4	4	
TNBC	11	13	11	5	
ER status					
ER positive	37	40	37	18	0.95
ER negative	17	20	14	9	
PgR status					
PgR positive	27	29	26	13	0.99
PgR negative	25	28	22	13	
No data	2	3	3	1	
HER2 status					
0	17	17	13	8	0.99
1+	16	19	18	10	
2+	13	15	15	4	
3+	8	9	5	5	
Grade					
Grade 1	4	4	4	0	0.53
Grade 2	22	24	21	10	
Grade 3	26	30	24	17	
No data	2	2	2	0	

a Kruskal–Wallis test.

### 2.3 Preparation of breast TIF and NIF samples

TIF and NIF samples were extracted from surgically resected small pieces of breast tumor and normal tissue that were collected as mastectomy specimens as previously described (Celis *et al*., 2004). Briefly, each resected, clean fresh tissue sample (0.2–0.3 g, as it was evaluated in our previous study) (Celis *et al*., 2004) was cut into small pieces (1 mm^3^), washed twice with cold PBS to remove traces of blood and cell debris, and placed in a 10‐mL conical plastic tube containing 1.0 mL of PBS. Samples were incubated for 1 h at 37 °C in a humidified CO_2_ incubator. Thereafter, the samples were centrifuged at 300 ***g*** for 2 min, and the supernatant was aspirated with the aid of an elongated Pasteur pipette. Samples were further centrifuged at 4000 ***g*** for 20 min in a refrigerated centrifuge (4 °C). These sequential centrifugations greatly facilitate removal of debris. Supernatants were collected and total protein concentrations for each sample were determined with the Bradford assay (Bradford, 1976). Only tumor specimens with 0.2–0.3 g were used for TIF recovery.

### 2.4 microRNA profiling

Due to the low abundance of microRNAs in the NIF and serum samples, TaqMan® Low Density Arrays (TLDA, cat# 4444913; Applied Biosystems, Foster City, CA, USA) were used. A total of 754 unique microRNAs were analyzed in the 60 TIF, 51 NIF, and 27 matched serum samples. Additionally, 54 tumor samples and corresponding TIFs isolated from 14 of these tumor samples were selected to undergo further analysis on high‐density hybridization arrays with 2549 human microRNAs represented (based on miRBase database release 21, Human microRNA Microarrays, 8x60K, v.21, G4872A; Agilent Technologies, Santa Clara, CA, USA). For details, see Doc S1.

### 2.5 Data normalization

Data generated from the TLDA cards were normalized per sample using global normalization (Mestdagh *et al*., 2009), and the expression of each microRNA was mean‐centered using only expressed microRNAs. Nondetermined values were replaced with the lowest value detected on a linear scale (Stahlberg *et al*., 2013). For normalization of the data generated from the hybridization arrays, the 90th percentile was calculated for each array, and then, this value was subtracted from each value on the array before baseline correction and a log2 transformation were applied (Gene Spring, v.13 Agilent Technologies). For details, see Doc S1.

### 2.6 Statistical analysis

r version 3.2.1 was utilized for statistical analyses. The Wilcoxon rank‐sum test was used for testing differential expression, and a false discovery rate (FDR) < 0.01 was considered significant. Multiple comparisons between subtypes were made using the Kruskal–Wallis analysis of variance (ANOVA) test for the identification of differentially expressed microRNAs across the subtypes. Univariate and multivariate regression analyses were performed to evaluate associations between CD3^+^, CD4^+^, CD45^+^, CD8^+^, and CD68^+^ lymphocytes, adipocytes, and the level of microRNAs in TIF. Spearman's rank correlation analysis was used to assess the association between microRNA levels across different cellular components. The log‐rank test with Kaplan–Meier plots was used for univariate survival analysis, while Cox regression was conducted for multivariate survival analysis. The differential abundance of 61 microRNAs detected in serum was validated in an independent cohort of Chinese patients with breast cancer (*n* = 32) and healthy controls (*n* = 22) that were retrieved from Gene Expression Omnibus (accession number: GSE42128) (Chan *et al*., 2013). Unsupervised hierarchical clustering was performed using Spearman's rank correlation analysis and average linkage in jexpress (Dysvik and Jonassen, 2001). For pathway analysis, QIAGEN's Ingenuity^®^ Pathway Analysis (QIAGEN, Redwood City, www.qiagen.com/ingenuity) was utilized. A FDR < 0.05 was considered significant. More details are provided in Doc S1.

## 3 Results

### 3.1 Comparative microRNA profiling of TIF, NIF, serum, and tumor tissue samples

The numbers of microRNAs present in more than 30% of the TIF, NIF, and corresponding serum samples were 457, 184, and 201, respectively (Fig. S2). Two microRNAs were detected exclusively in the NIF samples, eight microRNAs were unique to the serum samples, and 218 microRNAs were only detected in the TIF samples. A total of 343 microRNAs were present in more than 30% of all three sample types (data not shown).

To compare the microRNA profiles of the tumor, TIF, NIF, and serum samples, Spearman's rank correlation analysis was performed. Because the correlation of low abundant microRNAs was also of interest, none of the microRNAs were filtered out (*n* = 754 in TLDA and *n* = 730 in hybridization arrays). The highest number of correlated microRNAs was found between the tumor and TIF samples (*n* = 62) and between the TIF and NIF samples (*n* = 61). Fewer microRNAs correlated with serum (*n* = 48 for TIF and *n* = 39 for NIF). All of the microRNAs that exhibited a significant correlation (*P* < 0.05) between the different sample types are listed in Table S4.

### 3.2 Detection of 61 unique microRNAs in TIF samples: candidate breast cancer biomarkers in serum

**Figure 1:**
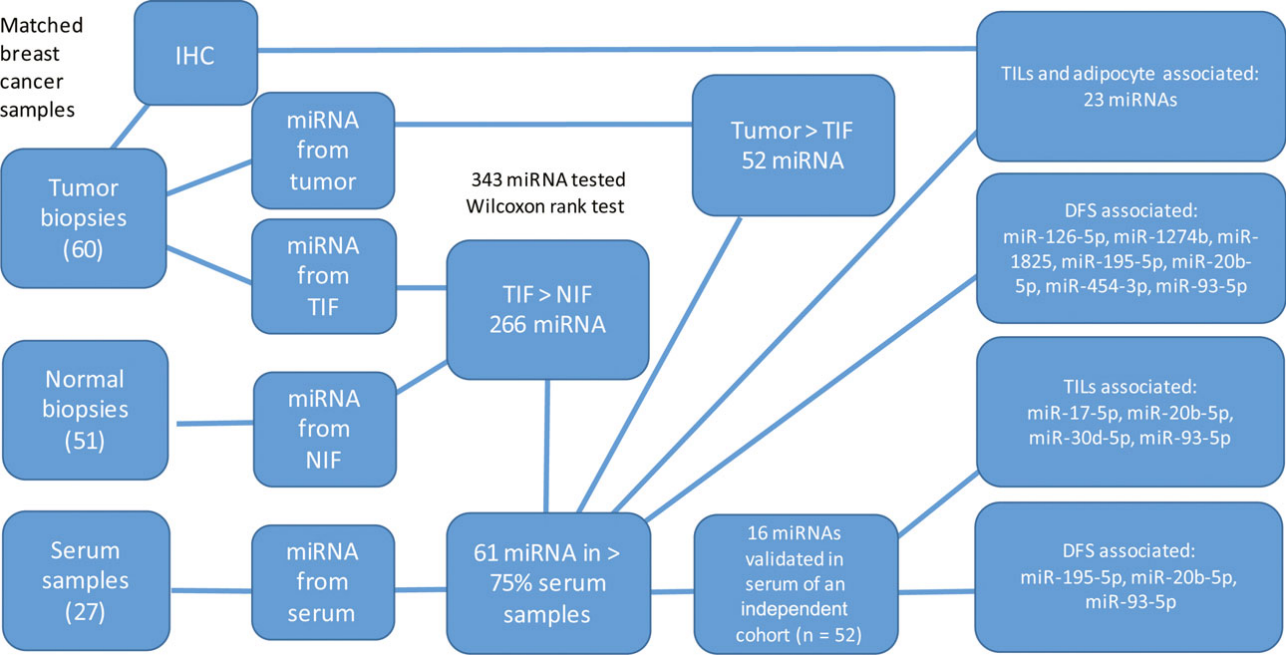
Flowchart of the strategy used to identify breast cancer biomarkers. First, microRNAs with significantly higher abundance in TIF samples compared to NIF samples were identified (*n* = 266). Of these, 61 microRNAs were detected in > 75% of serum samples and are referred to as potential biomarker candidates. Fifty‐two of the 61 candidates exhibited a higher abundance in tumor tissue samples than in TIF samples. In addition, 16 of these 52 microRNAs had a higher abundance in the serum of patients with breast cancer compared to controls in an independent dataset. In the two upper right panels, the expression levels of the 61 candidates identified in the TIF samples were examined for their association with TILs, adipocytes, and DFS. Twenty‐three microRNA candidates were associated with tumor infiltration of TILs or the presence of adipocytes, while seven microRNAs were associated with DFS. In the two lower right panels, the 16 validated microRNAs were associated with cellular components within a tumor mass (TILs) and were correlated with DFS. TILs, tumor‐infiltrating lymphocytes; DFS, disease‐free survival; NIF, normal interstitial fluid; TIF, tumor interstitial fluid.

To identify tumor‐associated biomarkers in serum, we selected microRNAs present in high abundance in TIF samples relative to NIF samples and with measurable levels in serum. The Wilcoxon rank‐sum test identified 266 microRNAs with a significantly higher abundance in TIF samples relative to NIF samples (FDR < 0.01, Table S5). Of these, 61 microRNAs were detected in more than 75% of the serum samples (Table S6 and Doc S1), and these represent potential serum biomarker candidates. An overview of the biomarker search that was conducted is presented in Fig. 1.

To reveal a possible association between the 61 microRNAs that were detected in TIF and serum samples with their corresponding intracellular levels, we compared the microRNA profiles of tumor samples and matched TIF samples on hybridization arrays. Three of the 61 microRNAs were not included on the array and six of the microRNAs did not significantly differ. However, 52 of the microRNAs assayed were significantly elevated in the tumor samples compared with the TIF samples (FDR < 0.01, Table S7).

**Figure 2:**
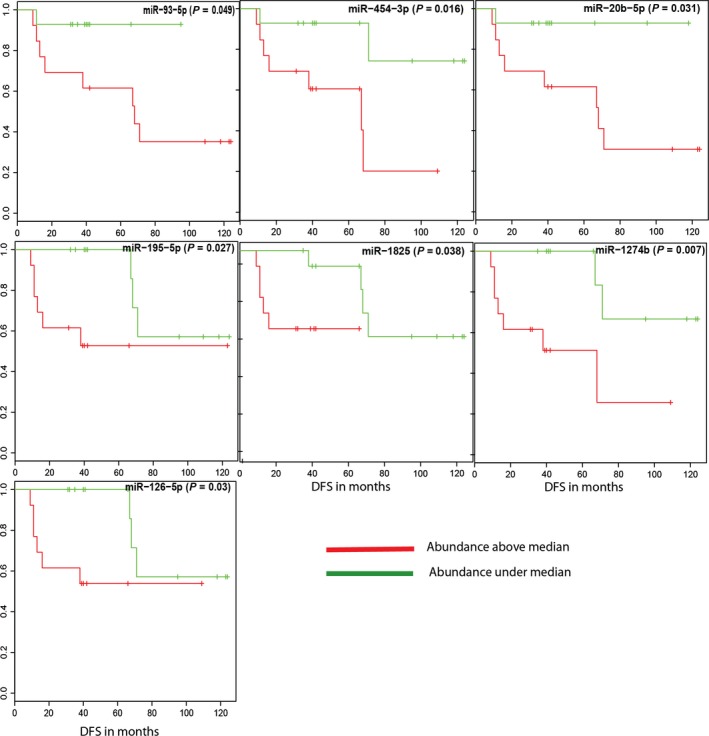
Kaplan–Meier curves for microRNAs whose abundance in serum was associated with time to recurrence. The green and red curves represent the abundance under and above the median, respectively.

To evaluate the potential clinical relevance of these 61 microRNAs identified as candidates for a blood‐based prognostic test, Kaplan–Meier survival analyses were performed for all of the serum samples to identify microRNAs that might correlate with DFS. Of the 61 candidates present in the serum samples, seven of the microRNAs (miR‐126‐5p, miR‐1274b, miR‐1825, miR‐195‐5p, miR‐20b‐5p, miR‐454‐3p, and miR‐miR‐93‐5p) were found to be significantly related to DFS (Fig. 2). Because our dataset did not include samples from healthy controls, all 61 microRNAs were evaluated in an independent Asian breast cancer serum cohort (Chan *et al*., 2013). Of the 61 candidates, nine of the microRNAs were not assessed in the Asian dataset. Of the 52 remaining microRNAs, 16 (miR‐16, miR‐17, miR‐195, miR‐19a, miR‐20b, miR‐210, miR‐25, miR‐30a, miR‐30d, miR‐320a, miR‐378, miR‐425, miR‐484, miR‐574‐3p, miR‐92a, and miR‐93) were significantly more abundant in the serum of patients with breast cancer relative to the healthy controls (FDR < 0.05, Table S8). For visualization, the relative abundance of the 16 validated microRNAs in TIF, NIF, and serum samples is illustrated with a heatmap in Fig. S3. Three of the microRNAs, miR‐195‐5p, miR‐20b‐5p, and miR‐93‐5p, were also associated with DFS when analyzed in our cohort (Figs 1 and 2).

### 3.3 microRNAs in the tumor interstitium are associated with breast tumor subtype and TILs

**Figure 3:**
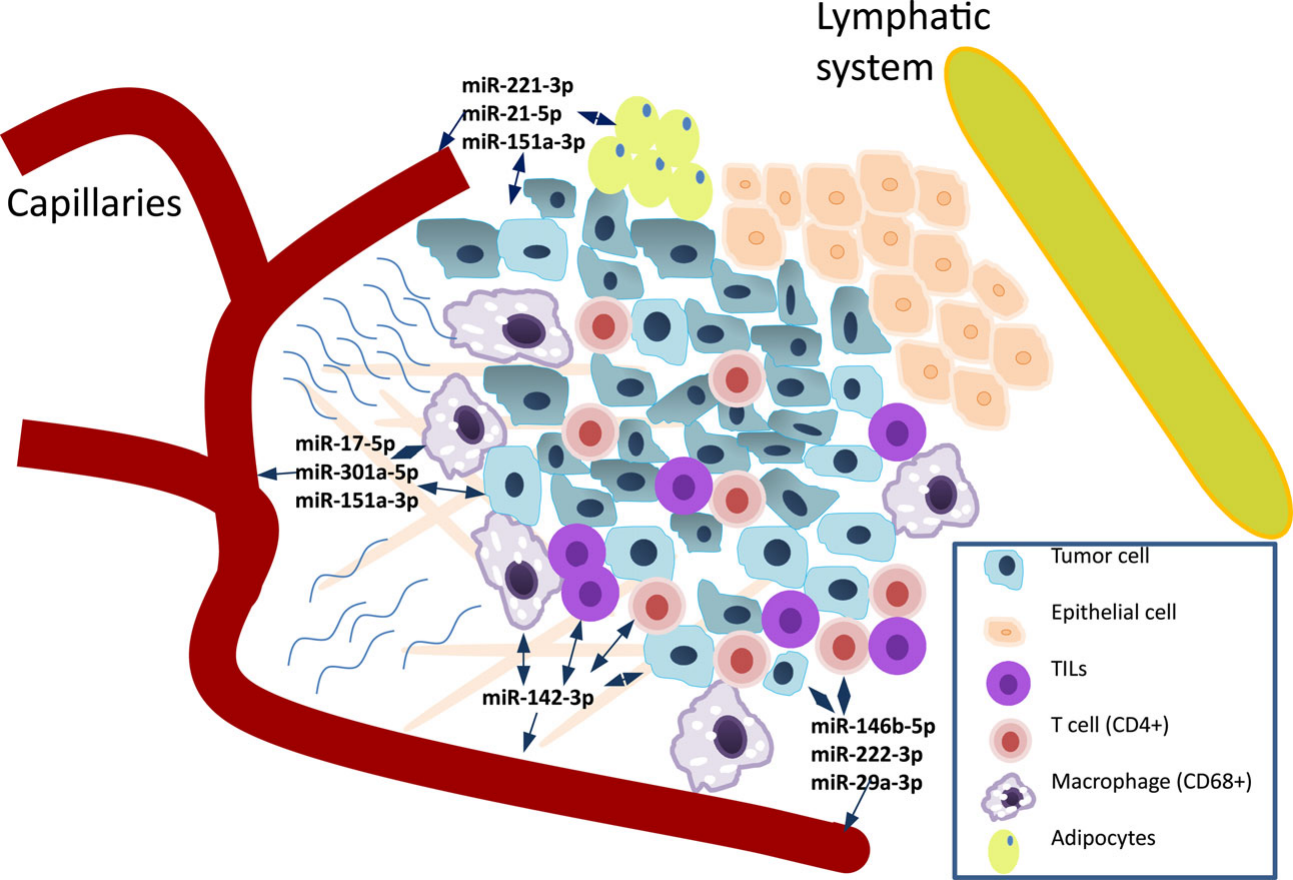
microRNAs in TIF and serum that correlate with the presence of immune cells, adipocytes, and tumor cells. These microRNAs are most likely involved in the cross‐talk between tumor cells and the microenvironment. TIF, tumor interstitial fluid.

To elucidate the cellular origin of the microRNAs identified in the TIF samples, a multivariate regression analysis was conducted to examine the proportion of tumor cells, the presence of adipocytes, and the presence of various TIL subtypes associated with each TIF microRNA. Of the 457 microRNAs that were analyzed, the proportion of tumor cells and the presence of TILs were two factors that significantly contributed to the variations observed for 179 of these microRNAs (FDR < 0.05, Table S9). In a univariate regression analysis, the proportion of TILs and the presence of adipocytes were the factors that significantly contributed to the variations observed in 23 of the 61 microRNA candidates examined (Table 2 and Fig. 1). For miR‐142‐3p, 28% of its variation could be explained by the presence of TILs. Moreover, depending on the type of lymphocytes present, the range of variation was 16–23%. The levels of mir‐454‐3p, miR‐20b‐5p, and miR‐93‐5p significantly correlated with the presence of TILs. Furthermore, high levels of these microRNAs in serum were associated with a worse prognosis (Fig. 1). Regarding the presence of miR‐93‐5p in the TIF samples, its levels significantly correlated with its expression in tumors and with the presence of adipocytes within a tumor mass. A possible scenario for microRNA interactions between tumor cells and stromal cells is shown in Fig. 3. Four microRNAs correlated with the presence of TILs and also exhibited significantly higher expression levels in the serum of patients with breast cancer relative to healthy controls. These microRNAs were miR‐17, miR‐20b, miR‐30d, and miR‐93 (Fig. 1).

**Table 2:** Univariate regression analysis revealed that the presence of TILs and adipocytes significantly contributed to the variation observed for 23 microRNAs in an independent manner. The microRNAs are listed alphabetically. Antibodies against CD45^+^, CD3^+^, CD4^+^, CD8^+^, and CD68^+^ were used to determine total leukocytes, T lymphocytes, T‐helper lymphocytes, cytotoxic T lymphocytes, and macrophages, respectively. TILs, tumor‐infiltrating lymphocytes; ns, not significant. Values with *P* < 0.05 (*P*) are listed in the table with their Spearman's rank correlation values (*R*). *R*‐values ≥ 0.20 are shown in boldface

miRNA	TIL		CD 68		CD 8		CD 4		CD 3		Adipose	
	*P*‐value	R	*P*‐value	R	*P*‐value	R	*P*‐value	R	*P*‐value	R	*P*‐value	R
let‐7e‐5p	0.05	0.06	ns	ns	ns	ns	ns	ns	ns	ns	ns	ns
miR‐140‐5p	ns	ns	ns	ns	ns	ns	0.03	0.17	ns	ns	ns	ns
miR‐142‐3p	0.00	**0.28**	0.02	**0.20**	0.03	0.18	0.01	**0.23**	0.05	0.16	ns	ns
miR‐146a‐5p	0.03	0.08	ns	ns	ns	ns	ns	ns	ns	ns	ns	ns
miR‐146b‐5p	0.00	0.19	ns	ns	ns	ns	0.02	0.19	ns	ns	ns	ns
miR‐151a‐3p	0.03	0.08	0.02	**0.20**	ns	ns	ns	ns	ns	ns	0.00	**0.24**
miR‐17‐5p	0.01	0.10	0.02	**0.20**	ns	ns	ns	ns	0.02	0.19	ns	ns
miR‐20b‐5p	0.03	0.08	ns	ns	ns	ns	ns	ns	ns	ns	ns	ns
miR‐21‐5p	ns	ns	ns	ns	ns	ns	ns	ns	ns	ns	0.03	0.18
miR‐221‐3p	ns	ns	ns	ns	ns	ns	ns	ns	ns	ns	0.01	**0.23**
miR‐222‐3p	0.04	0.07	ns	ns	0.04	0.16	0.01	**0.21**	ns	ns	ns	ns
miR‐223‐3p	0.00	0.14	ns	ns	ns	ns	ns	ns	0.03	0.17	ns	ns
miR‐223‐5p	0.01	0.13	ns	ns	ns	ns	ns	ns	0.04	0.17	ns	ns
miR‐27a‐3p	0.00	0.17	0.05	0.16	ns	ns	ns	ns	ns	ns	ns	ns
miR‐29a‐3p	ns	ns	ns	ns	ns	ns	0.02	**0.20**	ns	ns	ns	ns
miR‐30d‐5p	ns	ns	ns	ns	ns	ns	ns	ns	ns	ns	0.04	0.17
miR‐301a‐5p	0.05	0.06	0.02	**0.20**	ns	ns	ns	ns	ns	ns	ns	ns
miR‐324‐3p	ns	ns	ns	ns	ns	ns	ns	ns	ns	ns	0.04	0.17
miR‐345‐5p	0.04	0.07	ns	ns	ns	ns	ns	ns	ns	ns	0.05	0.16
miR‐374a‐5p	0.02	0.09	ns	ns	ns	ns	ns	ns	ns	ns	ns	ns
miR‐454‐3p	0.01	0.13	0.03	0.18	ns	ns	ns	ns	ns	ns	ns	ns
miR‐885‐5p	ns	ns	ns	ns	ns	ns	0.03	0.18	ns	ns	ns	ns
miR‐93‐5p	0.04	0.07	ns	ns	ns	ns	ns	ns	ns	ns	0.04	0.16

To identify pathways potentially altered by the microRNAs that positively correlated with the presence of TILs, an ingenuity pathway analysis was conducted. Pathway analyses were also conducted for the microRNAs that correlated with an infiltration of adipocytes and specific types of lymphocytes (including CD3^+^, CD4^+^, CD45^+^, CD8^+^, and CD68^+^ lymphocytes) (Table S10). The pathway, ‘Molecular Mechanism of Cancer’, was found to be altered for the microRNAs that correlated with the presence of CD3^+^, CD45^+^, CD8^+^, and CD68^+^ lymphocytes as well as infiltrating adipocytes, but not CD4^+^ lymphocytes.

**Figure 4:**
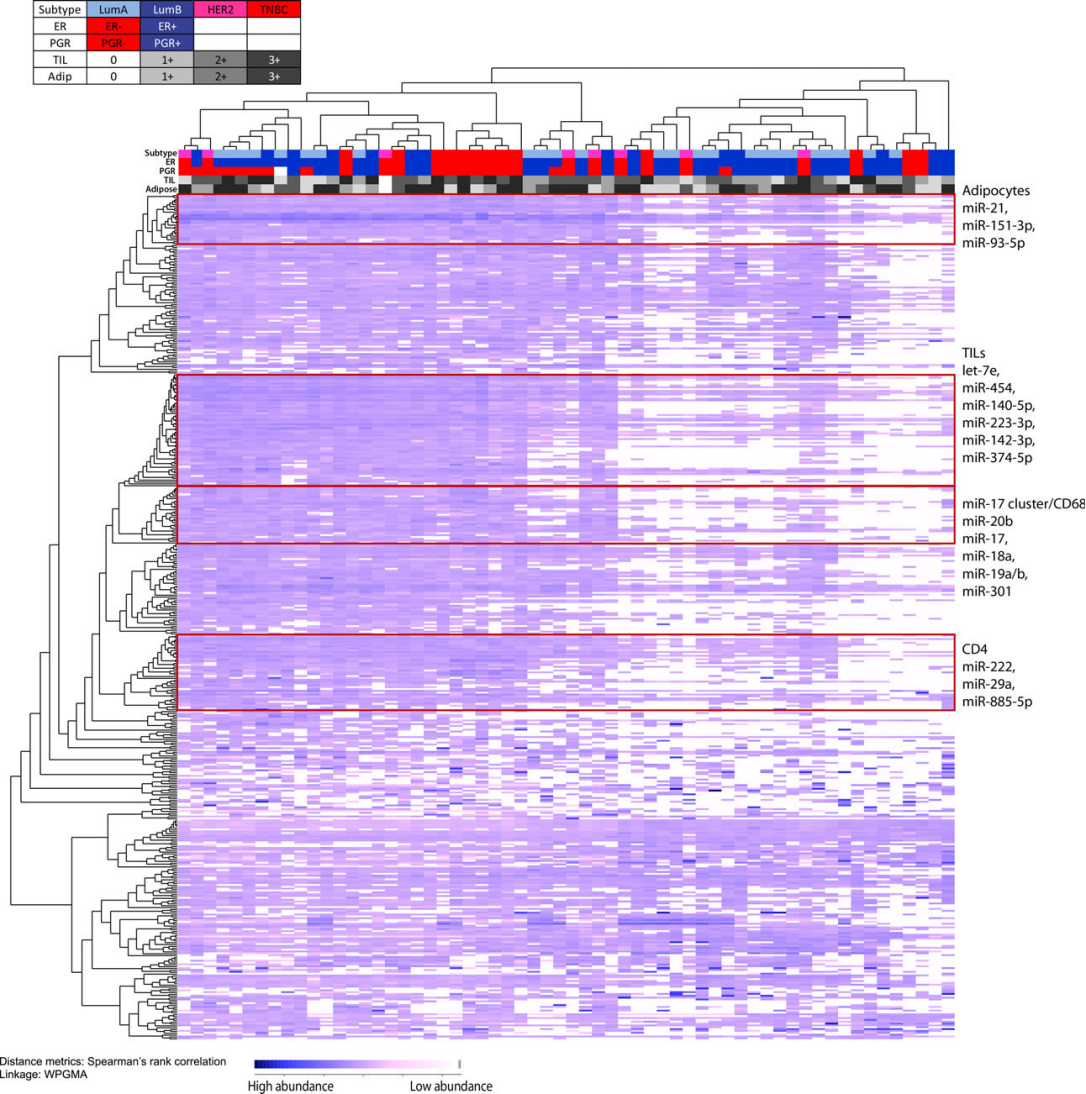
Unsupervised hierarchical clustering of microRNA abundance in 60 TIF samples resulted in the identification of two main clusters. Clinical parameters are indicated to the left of each cluster. In the heatmap, high abundance and low abundance of microRNAs are represented with dark blue and pink colorings, respectively. Missing values are shown in white, indicating that the microRNA was not detected in the TIF sample. Four subclusters enriched with microRNAs associated with TILs and adipocytes were identified and are indicated with a red frame. The microRNAs within the four clusters are listed to the right.

To study whether a correlation exists between TIF microRNAs and parameters such as subtype and hormone receptor status, unsupervised hierarchical clustering with Spearman's rank correlation and average linkage was performed for all 457 microRNAs (Fig. 4). The 60 TIF samples clustered into two main groups. The TNBC samples primarily clustered into one subgroup of the first main cluster. The luminal A samples were distributed between both clusters. A greater number of ER‐ and PgR‐negative samples, as well as a higher proportion of TILs, were present in cluster 1. Furthermore, according to the log‐rank test, patients in cluster 1 experienced a longer DFS period (*P* = 0.058) (Fig. S4A). When the data were stratified into subtypes, only the luminal A subtype exhibited a significant better survival in cluster 1 (Fig. S4B). The parameters, tumor grade, patient age, and tumor stage and size did not significantly differ between clusters 1 and 2. However, a tendency for grade 3 samples to be in cluster 1 was observed (*P* = 0.082, logistic regression).

In the two main microRNA clusters that were identified, there were several subclusters of microRNAs within each. Four of the microRNA subclusters were enriched with microRNAs associated with adipocyte‐, TIL‐, CD68‐, and CD4‐associated microRNAs. The microRNA cluster that was associated with CD68^+^ cells was enriched with microRNAs from the miR‐17‐92 family.

To identify subtype‐specific microRNAs across the TIF samples, the Kruskal–Wallis test and Wilcoxon rank‐sum test were performed for all 457 microRNAs (*P* < 0.05, Tables 3 and S11). Three of the microRNAs (miR‐190b, miR‐375, and miR‐376a‐5p) had an FDR < 0.05 and four microRNAs (miR‐190b, miR‐224‐5p, miR‐342‐3p, and miR‐376a‐5p) exhibited significantly different levels in luminal samples versus HER2‐positive and TNBC samples. In addition, compared with all other subtypes, miR‐671‐3p and 342‐5p showed significantly different abundance in HER2‐positive samples, miR‐1281 had a lower level in the luminal A samples, and miR‐524‐3p was found at lower level in the luminal B samples. Four of the subtype‐specific microRNAs detected in TIF (miR‐342‐3p, miR‐342‐5p, miR‐224‐5p, and miR‐375) showed positive correlation with the abundance of the same microRNA in the tumor samples. One of these (miR‐342‐3p) was correlated with the level in serum as well. Two (miR‐375 and miR‐27a‐5p) of the microRNAs with high abundance in the luminal A and HER2‐positive samples were associated with poor prognosis.

**Table 3:** microRNAs (*n* = 12) in TIF significantly associated with breast cancer subtypes. Higher abundance of miR‐27a‐5p and miR‐375 was associated with shorter DFS periods. DFS, disease‐free survival; TIF, tumor interstitial fluid

	LumA	LumB	HER2	TNBC
miR‐1282	Down	Up	Up	Up
miR‐190b	Up^a^	Up	Down	Down^a^
miR‐224‐5p	Down	Down	Up	Up
miR‐27a‐5p^b^	Up	Down	Up	ns
miR‐342‐3p	Up	Up	Down	Down
miR‐342‐5p	Up	Up	Down	Up
miR‐375^b^	Up^a^	ns	Up	Down^a^
miR‐376a‐5p	Up^a^	Up	Down	Down^a^
miR‐432‐3p	Down	Up	Down	Up
miR‐524‐3p	Up	Down	Up	Up
miR‐671‐3p	Down	Down	Up	Down
miR‐9‐5p	Down	Down	ns	Up

a FDR < 0.05.

b High level associated with decreased DFS.

## 4 Discussion

### 4.1 Identification of putative diagnostic and prognostic microRNA biomarker candidates for breast cancer

To our knowledge, this is the first study to investigate microRNAs present in TIF with the goal of identifying noninvasive diagnostic markers for breast cancer. A total of 61 microRNAs were present in greater abundance in TIF samples than in NIF samples and were also present in > 75% of the serum samples. Higher levels of expression were also observed for 51 of these 61 candidates in tumor samples compared to TIF samples, thereby supporting the hypothesis that these are tumor‐derived microRNAs. These findings confirm the concept that fluid proximal to a primary tumor is a promising source for disease biomarkers, and they also demonstrate the ability of our approach to identify potential serum biomarkers of breast cancer. Further, the low number of correlated microRNAs between tumor and TIF supports that TIF contains mainly secreted miRNAs without major contamination from intracellular molecules. This is previously demonstrated at the protein level, where mass spectrometry showed absence of intracellular proteins like keratins and nuclear proteins (Celis *et al*., 2004; Gromov *et al*., 2010). To validate these data, 52 of the 61 microRNAs were tested against an independent set of serum samples obtained from a Chinese cohort of patients with breast cancer and healthy individuals. Sixteen of the microRNAs were subsequently identified as potential noninvasive breast cancer biomarkers. Interestingly, several of these 16 validated microRNAs have previously been proposed to represent prognostic or diagnostic biomarkers for breast cancer. These include miR‐210 (Volinia *et al*., 2012), miR‐195 (Heneghan *et al*., 2010), miR‐20b (Zhou *et al*., 2014), and miR‐484 (Zearo *et al*., 2014). Our results also showed that high levels of miR‐93, miR‐195, and miR‐20b were associated with poor DFS, while the levels of miR‐20b and miR‐93 in the TIF samples correlated with the presence and type of TILs present. In general, TILs are accepted as prognostic factors for breast cancer (Savas *et al*., 2015). The importance of TILs and other components of breast cancer microenvironment as clinically relevant biomarkers reinforces by an International TILs Working Group created in 2014 (Salgado *et al*., 2015). However, as a relatively small number of matched serum samples (*n* = 27) were included in the present study, further studies need to be conducted with larger groups of patients to confirm these findings. In addition, it is possible that the small number of matched serum samples prevented the identification of other relevant microRNAs.

### 4.2 microRNAs in the interstitium are associated with patient survival and reflect breast cancer subtypes

Unsupervised hierarchical clustering revealed two main clusters for the TIF samples examined in this study. Cluster 1 was enriched with hormone receptor‐negative (e.g., ER‐, PgR‐, and HER2‐) and samples with high TILs, which suggests that microRNAs detected in the interstitium are associated with breast tumor subtype, as well as immune response. Interestingly, cluster 1 also revealed a higher abundance of the microRNAs. This was especially pronounced for microRNAs associated with the presence of TILs. This is in line with the growing evidence of immunology as a central part of the tumor biology and outcome (Salgado *et al*., 2015). The two main sample clusters that were identified represented different disease courses, and a significantly better outcome was observed for the patients in cluster 1. These data imply that microRNAs that are detected in the TIF and originate from both cancer cells and stromal cells may reflect biological processes that are associated with prognosis. Unexpectedly, however, cluster 1 also included a subcluster of TNBC samples, and the association between TNBC tumors and poor prognosis is well known. This subcluster of samples was also associated with a high number of TILs, which is a characteristic often associated with improved survival, especially in TNBC‐ and HER2‐positive patients (Savas *et al*., 2015). The difference in survival between the two clusters was particularly pronounced for the luminal A samples. In particular, two microRNAs, miR‐27a‐5p and miR‐375 whose overexpression was related to unfavorable survival, exhibited significantly higher levels in luminal A and HER2‐positive samples relative to luminal B and TNBC samples. In addition, the level of miR‐375 in TIF was found to positively correlate with the percentage of a tumor, yet the same correlation was not observed for serum samples. In a study of metastatic breast cancer, high levels of miR‐375 in serum were significantly correlated with poor survival (Madhavan *et al*., 2016). Due to the small number of samples in each subgroup, our survival analysis should be interpreted with caution and additional studies with a greater number of samples are needed to further investigate these findings.

Recently, a number of specific microRNAs have been reported elevated in breast carcinomas (van Schooneveld *et al*., 2015), but not previously been investigated in interstitial fluids. In the present study, miR‐190b was found at higher levels in luminal A and luminal B samples compared with HER2 and TNBC samples, while TNBC samples contained higher levels of miR‐9‐5p compared with the luminal samples. Previously, lower levels of miR‐190b and higher levels of miR‐9 were detected in TNBC tumor biopsies (Volinia *et al*., 2012). Similarly, another study reported that miR‐190b was up‐regulated in luminal tumor samples compared with TNBC and HER2 tumor samples, and miR‐9 was only expressed in TNBC tumor samples and not in other subtype samples (Enerly *et al*., 2011). The level of miR‐190b and miR‐9 TIF did not correlate with the level detected in tumor in our project. On the other hand, four of the subtype‐specific microRNAs detected in TIF were correlated with the level in tumor tissue (miR‐342‐3p, miR‐342‐5p, miR‐224‐5p, and miR‐375). miR‐342‐3p was found more abundant in the TIF of luminal samples compared to HER2 and TNBC. In addition, the distribution was correlated both in the tumor samples and in the serum samples. Taken together, these results indicate that only a portion of the subtype‐specific microRNAs produced in a tumor may be released into the interstitium. Further, very few of these specific microRNAs can be detected in serum.

### 4.3 The presence of TILs, adipocytes, and tumor cells affects microRNAs

Of all the microRNAs detected in the TIF samples (*n* = 457), 179 were present at levels that were associated with the type of TILs present and the proportion of tumor cells in the corresponding tumor sample. Among the 61 candidate biomarkers identified, the presence of TILs or adipocytes influenced the variation observed for 23 of these microRNAs. When hierarchical clustering of the TIL‐associated microRNAs was performed, the subgroups identified were found to be enriched with microRNAs associated with CD4^+^ (used as a marker for T‐helper lymphocytes) and CD68^+^ (a marker for macrophage) cells (Fig. 4). In particular, miR‐223 was significantly correlated with CD3^+^ (a general marker of lymphocytes) and CD45^+^ (a pan‐leukocyte marker) cells. In another study, miR‐223 was detected in exosomes released from macrophages that were shuttled to co‐cultivated breast cancer cells, and this resulted in an *in vitro* invasion of breast cancer cells (Yang *et al*., 2011). miR‐223, miR‐146, let‐7e, and miR‐21 have also been identified as key factors for macrophage activation (Squadrito *et al*., 2013). Macrophages can be classified as classically activated (M1) or alternatively activated (M2). The latter promote an anti‐inflammatory response (Noy and Pollard, 2014) and are also referred to as TAMs. In a recent study of breast cancer, miR‐223 and miR‐146a were present at lower levels in TAMs, which suggested that these microRNAs play a role in regulating M2 polarization (Li *et al*., 2015). In the present study, miR‐142‐3p expression is highly correlated with TILs and with each subgroup of CD3^+^, CD4^+^, CD45^+^, CD68^+^, and CD8^+^ cells (with CD8^+^ used as a marker of cytotoxic T lymphocytes). These results are consistent with a report where miR‐142‐3p is highly expressed in T cells (Wu *et al*., 2007). Furthermore, up‐regulation of miR‐142‐3p has been reported to impair macrophage differentiation and to increase CD8^+^ cell proliferation, which resulted in less immunosuppressive activity (Sonda *et al*., 2013). It would be interesting to investigate which miRNAs are specifically originated from different TILs subtypes; however, such a project represents an independent and rather extensive study and is therefore beyond the scope of the present study.

The miR‐17/92 cluster, which is comprised of miR‐17, miR‐18a, miR‐19a, miR‐19b, miR‐20a, and miR‐92, is probably one of the most studied onco‐miR clusters to date (Mogilyansky and Rigoutsos, 2013). miR‐17 has been found to regulate the proliferation of CD8^+^ T cells (Khan *et al*., 2013) and to play a role in macrophage infiltration and cytokine secretion (Zhu *et al*., 2013). In the present study, CD68‐associated microRNAs were identified in the same cluster as the 17/92 onco‐miRs. In addition, miR‐17 positively correlated with the presence of CD68^+^ cells. miR‐17, miR‐19a, and miR‐92 were also among the 61 microRNA candidates that were validated in an independent breast cancer cohort. Based on these data, we hypothesize that a subset of the 16 validated microRNAs in serum are directly involved in the interplay between tumor cells and immune cells. Moreover, we find that several of the TIL‐correlated microRNAs identified (see Table 2) appear to have an active role in either pro‐inflammatory or anti‐inflammatory responses, and this reflects the communication that occurs between compartments present in the tumor stroma. Correspondingly, cancer‐associated cells and immune cells have previously been shown to contribute to a reactive stroma in response to a tumor (Tjomsland *et al*., 2011).

The pathway termed, ‘Molecular Mechanisms of Cancer’, by QIAGEN's Ingenuity^®^ Pathway Analysis program includes molecular components that potentially mediate inter‐ and intracellular communication that results in a malignant phenotype. This pathway was predicted to be affected by the presence of microRNAs that correlate with CD3^+^, CD45^+^, CD8^+^, and CD68^+^ lymphocytes, as well as infiltrating adipocytes. Similarly, microRNAs associated with TILs and CD4^+^ cells were enriched within the pathway termed, ‘Role of Macrophage, Fibroblasts, and Endothelial Cells in Rheumatoid Arthritis’. This pathway involves the activation of T cells, synovial fibroblasts, and macrophage, which leads to altered T‐cell and B‐cell signaling. The top canonical pathways predicted to be altered were similar for many of the TIL subgroups and this may be because these pathways are associated with many of the microRNAs that exhibited a higher abundance in the TIF samples compared with the NIF samples. Moreover, these abundant microRNAs may participate in some of the same processes encompassed by these pathways.

### 4.4 microRNAs present in TIF and serum samples as candidate blood‐based biomarkers

In the present study, the levels of microRNAs that were detected in the TIF and NIF samples were very different. Moreover, only a subset of the microRNAs with high abundance in the TIF samples were also detected in serum. The latter observation highlights the challenges associated with identifying blood‐based biomarkers, particularly because microRNAs derived from tumor cells are diluted in blood which also carries molecules from other cells in the body. For example, blood cells contribute microRNAs to the systemic circulation, and it has been suggested that the majority of microRNAs found in serum derive from platelets undergoing activation (Gyorgy *et al*., 2011). Thus, in the present study, we focused on detecting microRNAs present in TIF and serum in order to identify tumor‐related serum microRNAs. However, we anticipated that this subset of microRNAs would be diluted in serum and therefore would not be readily detected. To address this limitation, it may be important to use several microRNAs as biomarkers in order to strengthen the predictive power (Cui *et al*., 2015). So far 16 microRNAs were identified as putative blood‐based markers of breast cancer in the present study.

It is also important to note that these 16 microRNAs were identified and validated despite the existence of factors that are not directly related to cancer, yet can affect microRNA detection. For example, the collection and processing methods used for samples, the type of analytical platform employed, normalization of data, tumor stage, tumor grade, and/or patient ethnicity can all affect the microRNA profile of blood samples (Cui *et al*., 2015). Thus, the 16 microRNAs identified and validated appear to be robust biomarkers and they should be further evaluated for their capacity to indicate the presence of breast cancer.

Given that the tumor microenvironment is composed of various cells with different origins, such as immunological cells, only a portion of detected microRNAs may derive directly from tumor cells. microRNAs can regulate numerous processes and affect different mRNAs. This makes the backtracking of microRNAs to specific cells particularly challenging. *In vitro* experiments could link individual miRNAs to specific cell types, but can hardly encompass the complexity of a living tumor. As a diagnostic tool, a panel of microRNAs will most likely increase the robustness of integrative molecular signatures that can be used as the diagnostic markers. It is also possible that some microRNAs remain associated with the local interstitium, while others are able to access the circulation. It has been demonstrated that microRNAs can be sorted for incorporation into exosomes by various mechanisms, and microRNAs such as miR‐142‐3p and miR‐320 are frequently found to enter exosomes (Zhang *et al*., 2015). Thus, such sorting signals may be essential for retaining microRNAs in the interstitium.

## 5 Conclusion

Based on the present results, we hypothesize that 61 of the microRNAs identified originate specifically from tumor cells and/or from tumor stroma and that these microRNAs have a high potential for forming blood‐based breast cancer biomarkers for disease detection. We were able to validate 16 of these microRNAs in an independent set of serum data obtained from a Chinese cohort of patients with breast cancer, thus confirming their potential as diagnostic biomarkers. We advocate that microRNAs released into the tumor interstitium in response to cross‐talk between malignant cells and TILs during breast cancer progression may be detected in the serum of patients with breast cancer and serve as diagnostic or prognostic biomarkers.

## Author contributions

VDH, IIG, PG, A‐LB‐D, ÅH, and ARH conceived and designed the project. ARH acquired the data. ARH, VS, VDH, IIG, and PG analyzed and interpreted the data. ARH and VHD wrote the manuscript. M‐LM‐T, VTW, and NB collected the material and participated in the evaluation of the data. All authors read and revised the manuscript critically and approved the final manuscript.

## Supporting information


**Fig. S1.** A representative example of the distribution of TILs that were detected in tumor biopsies based on HE and IHC staining.

**Fig. S2.** A Venn diagram represents the microRNAs that were detected in > 30% of the TIF (*n* = 457), NIF (*n* = 184), and serum (*n* = 201) samples.

**Fig. S3.** Hierarchical clustering of the 16 identified biomarker candidates.

**Fig. S4.** Kaplan–Meier survival plots and log‐rank test with *P*‐values for the two clusters of microRNA data.

**Doc. S1.** Methods.

**Table S1.** An average expression of Ki67 used for subtype estimation and the cutoff of KI67 positivity was assigned in accordance with the currently accepted criteria (Esposito *et al*., 2015) and intrinsic subtypes were assigned as shown in the table, where the luminal B subtype was divided in two according to HER2‐status.
**Table S2.** An overview of the samples included in the microRNA profiling.
**Table S3.** Antibodies used in this study.
**Table S4.** Spearman Rank Correlation identified microRNAs correlated between TIF, NIF, Serum and tumor. *P* < 0.05 is regarded as significant.
**Table S5.** Wilcoxon Rank test identified 266 microRNAs with significantly higher abundance in TIF relatively to NIF (FDR < 0.01).
**Table S6.** 61 microRNAs were identified using the criteria: Up in TIF vs NIF (FDR < 0.01) and expressed in more than 75% of serum samples.
**Table S7.** The difference in abundance of the 61 candidate microRNAs were tested using student's t‐test and 52 microRNAs showed significantly higher abundance in tumor mass vs TIF.
**Table S8.** MicroRNA profiling in serum of Chinese breast cancer patients.
**Table S9.** Out of the 457 microRNA in TIF, the presence of TILs and tumor percentage contributed significantly to the variation of 179 microRNAs (FDR < 0.05).
**Table S10.** Pathway analyses were performed for microRNAs significantly correlated with subgroups of TILs and adipocytes.
**Table S11.** Kruskal‐Wallis Anova test was performed to identify microRNAs with differential distribution between the subgroups.

## References

[mol212025-bib-0001] Bertoli G , Cava C and Castiglioni I (2015) MicroRNAs: new biomarkers for diagnosis, prognosis, therapy prediction and therapeutic tools for breast cancer. Theranostics 5, 1122–1143. 26199650 10.7150/thno.11543 PMC4508501

[mol212025-bib-0002] Bradford MM (1976) A rapid and sensitive method for the quantitation of microgram quantities of protein utilizing the principle of protein‐dye binding. Anal Biochem 72, 248–254. 942051 10.1016/0003-2697(76)90527-3

[mol212025-bib-0003] Celis JE , Gromov P , Cabezon T , Moreira JM , Ambartsumian N , Sandelin K , Rank F and Gromova I (2004) Proteomic characterization of the interstitial fluid perfusing the breast tumor microenvironment: a novel resource for biomarker and therapeutic target discovery. Mol Cell Proteomics 3, 327–344. 14754989 10.1074/mcp.M400009-MCP200

[mol212025-bib-0004] Chan M , Liaw CS , Ji SM , Tan HH , Wong CY , Thike AA , Tan PH , Ho GH and Lee AS (2013) Identification of circulating microRNA signatures for breast cancer detection. Clin Cancer Res 19, 4477–4487. 23797906 10.1158/1078-0432.CCR-12-3401

[mol212025-bib-0005] Cui Z , Lin D , Song W , Chen M and Li D (2015) Diagnostic value of circulating microRNAs as biomarkers for breast cancer: a meta‐analysis study. Tumour Biol 36, 829–839. 25296735 10.1007/s13277-014-2700-8

[mol212025-bib-0006] Dysvik B and Jonassen I (2001) J‐Express: exploring gene expression data using Java. Bioinformatics 17, 369–370. 11301307 10.1093/bioinformatics/17.4.369

[mol212025-bib-0007] Enerly E , Steinfeld I , Kleivi K , Leivonen SK , Aure MR , Russnes HG , Ronneberg JA , Johnsen H , Navon R , Rodland E *et al* (2011) miRNA‐mRNA integrated analysis reveals roles for miRNAs in primary breast tumors. PLoS One 6, e16915. 21364938 10.1371/journal.pone.0016915 PMC3043070

[mol212025-bib-0008] Esposito A , Criscitiello C and Curigliano G (2015) Highlights from the 14(th) St Gallen International Breast Cancer Conference 2015 in Vienna: dealing with classification, prognostication, and prediction refinement to personalize the treatment of patients with early breast cancer. Ecancermedicalscience 9, 518. 25932042 10.3332/ecancer.2015.518 PMC4404037

[mol212025-bib-0009] Frediani JN and Fabbri M (2016) Essential role of miRNAs in orchestrating the biology of the tumor microenvironment. Mol Cancer 15, 42. 27231010 10.1186/s12943-016-0525-3 PMC4882787

[mol212025-bib-0010] Gooden MJ , de Bock GH , Leffers N , Daemen T and Nijman HW (2011) The prognostic influence of tumour‐infiltrating lymphocytes in cancer: a systematic review with meta‐analysis. Br J Cancer 105, 93–103. 21629244 10.1038/bjc.2011.189 PMC3137407

[mol212025-bib-0011] Gromov P , Gromova I , Bunkenborg J , Cabezon T , Moreira JM , Timmermans‐Wielenga V , Roepstorff P , Rank F and Celis JE (2010) Up‐regulated proteins in the fluid bathing the tumour cell microenvironment as potential serological markers for early detection of cancer of the breast. Mol Oncol 4, 65–89. 20005186 10.1016/j.molonc.2009.11.003 PMC5527961

[mol212025-bib-0012] Gromov P , Gromova I , Olsen CJ , Timmermans‐Wielenga V , Talman ML , Serizawa RR and Moreira JM (2013) Tumor interstitial fluid – a treasure trove of cancer biomarkers. Biochim Biophys Acta 1834, 2259–2270. 23416532 10.1016/j.bbapap.2013.01.013

[mol212025-bib-0013] Gyorgy B , Szabo TG , Pasztoi M , Pal Z , Misjak P , Aradi B , Laszlo V , Pallinger E , Pap E , Kittel A *et al* (2011) Membrane vesicles, current state‐of‐the‐art: emerging role of extracellular vesicles. Cell Mol Life Sci 68, 2667–2688. 21560073 10.1007/s00018-011-0689-3 PMC3142546

[mol212025-bib-0014] Heneghan HM , Miller N , Kelly R , Newell J and Kerin MJ (2010) Systemic miRNA‐195 differentiates breast cancer from other malignancies and is a potential biomarker for detecting noninvasive and early stage disease. Oncologist 15, 673–682. 20576643 10.1634/theoncologist.2010-0103 PMC3228012

[mol212025-bib-0015] Kao J , Salari K , Bocanegra M , Choi YL , Girard L , Gandhi J , Kwei KA , Hernandez‐Boussard T , Wang P , Gazdar AF *et al* (2009) Molecular profiling of breast cancer cell lines defines relevant tumor models and provides a resource for cancer gene discovery. PLoS One 4, e6146. 19582160 10.1371/journal.pone.0006146 PMC2702084

[mol212025-bib-0016] Khan AA , Penny LA , Yuzefpolskiy Y , Sarkar S and Kalia V (2013) MicroRNA‐17~92 regulates effector and memory CD8 T‐cell fates by modulating proliferation in response to infections. Blood 121, 4473–4483. 23596046 10.1182/blood-2012-06-435412

[mol212025-bib-0017] Kohlhapp FJ , Mitra AK , Lengyel E and Peter ME (2015) MicroRNAs as mediators and communicators between cancer cells and the tumor microenvironment. Oncogene 34, 5857–5868. 25867073 10.1038/onc.2015.89 PMC4604012

[mol212025-bib-0018] Li F , Guo Z , Lizee G , Yu H , Wang H and Si T (2014) Clinical prognostic value of CD4+CD25+FOXP3+ regulatory T cells in peripheral blood of Barcelona Clinic Liver Cancer (BCLC) stage B hepatocellular carcinoma patients. Clin Chem Lab Med 52, 1357–1365. 24646790 10.1515/cclm-2013-0878

[mol212025-bib-0019] Li Y , Zhao L , Shi B , Ma S , Xu Z , Ge Y , Liu Y , Zheng D and Shi J (2015) Functions of miR‐146a and miR‐222 in tumor‐associated macrophages in breast cancer. Sci Rep 5, 18648. 26689540 10.1038/srep18648 PMC4686897

[mol212025-bib-0020] Ma L and Weinberg RA (2008) MicroRNAs in malignant progression. Cell Cycle 7, 570–572. 18256538 10.4161/cc.7.5.5547

[mol212025-bib-0021] Mackay A , Tamber N , Fenwick K , Iravani M , Grigoriadis A , Dexter T , Lord CJ , Reis‐Filho JS and Ashworth A (2009) A high‐resolution integrated analysis of genetic and expression profiles of breast cancer cell lines. Breast Cancer Res Treat 118, 481–498. 19169812 10.1007/s10549-008-0296-7

[mol212025-bib-0022] Madhavan D , Peng C , Wallwiener M , Zucknick M , Nees J , Schott S , Rudolph A , Riethdorf S , Trumpp A , Pantel K *et al* (2016) Circulating miRNAs with prognostic value in metastatic breast cancer and for early detection of metastasis. Carcinogenesis 37, 461–470. 26785733 10.1093/carcin/bgw008

[mol212025-bib-0023] Mesker WE , Junggeburt JM , Szuhai K , de Heer P , Morreau H , Tanke HJ and Tollenaar RA (2007) The carcinoma‐stromal ratio of colon carcinoma is an independent factor for survival compared to lymph node status and tumor stage. Cell Oncol 29, 387–398. 17726261 10.1155/2007/175276 PMC4617992

[mol212025-bib-0024] Mestdagh P , Van Vlierberghe P , De Weer A , Muth D , Westermann F , Speleman F and Vandesompele J (2009) A novel and universal method for microRNA RT‐qPCR data normalization. Genome Biol 10, R64. 19531210 10.1186/gb-2009-10-6-r64 PMC2718498

[mol212025-bib-0025] Mogilyansky E and Rigoutsos I (2013) The miR‐17/92 cluster: a comprehensive update on its genomics, genetics, functions and increasingly important and numerous roles in health and disease. Cell Death Differ 20, 1603–1614. 24212931 10.1038/cdd.2013.125 PMC3824591

[mol212025-bib-0026] Noy R and Pollard JW (2014) Tumor‐associated macrophages: from mechanisms to therapy. Immunity 41, 49–61. 25035953 10.1016/j.immuni.2014.06.010 PMC4137410

[mol212025-bib-0027] Perou CM , Sorlie T , Eisen MB , van de Rijn M , Jeffrey SS , Rees CA , Pollack JR , Ross DT , Johnsen H , Akslen LA *et al* (2000) Molecular portraits of human breast tumours. Nature 406, 747–752. 10963602 10.1038/35021093

[mol212025-bib-0028] Prat A and Perou CM (2011) Deconstructing the molecular portraits of breast cancer. Mol Oncol 5, 5–23. 21147047 10.1016/j.molonc.2010.11.003 PMC5528267

[mol212025-bib-0029] Riches A , Campbell E , Borger E and Powis S (2014) Regulation of exosome release from mammary epithelial and breast cancer cells – a new regulatory pathway. Eur J Cancer 50, 1025–1034. 24462375 10.1016/j.ejca.2013.12.019

[mol212025-bib-0030] Ruffell B , Au A , Rugo HS , Esserman LJ , Hwang ES and Coussens LM (2012) Leukocyte composition of human breast cancer. Proc Natl Acad Sci U S A 109, 2796–2801. 21825174 10.1073/pnas.1104303108 PMC3287000

[mol212025-bib-0031] Salgado R , Denkert C , Demaria S , Sirtaine N , Klauschen F , Pruneri G , Wienert S , Van den Eynden G , Baehner FL , Penault‐Llorca F *et al* (2015) The evaluation of tumor‐infiltrating lymphocytes (TILs) in breast cancer: recommendations by an International TILs Working Group 2014. Ann Oncol 26, 259–271. 25214542 10.1093/annonc/mdu450 PMC6267863

[mol212025-bib-0032] Savas P , Salgado R , Denkert C , Sotiriou C , Darcy PK , Smyth MJ and Loi S (2015) Clinical relevance of host immunity in breast cancer: from TILs to the clinic. Nat Rev Clin Oncol 13, 228–241. 26667975 10.1038/nrclinonc.2015.215

[mol212025-bib-0033] van Schooneveld E , Wildiers H , Vergote I , Vermeulen PB , Dirix LY and Van Laere SJ (2015) Dysregulation of microRNAs in breast cancer and their potential role as prognostic and predictive biomarkers in patient management. Breast Cancer Res 17, 21. 25849621 10.1186/s13058-015-0526-y PMC4332424

[mol212025-bib-0034] Sonda N , Simonato F , Peranzoni E , Cali B , Bortoluzzi S , Bisognin A , Wang E , Marincola FM , Naldini L , Gentner B *et al* (2013) miR‐142‐3p prevents macrophage differentiation during cancer‐induced myelopoiesis. Immunity 38, 1236–1249. 23809164 10.1016/j.immuni.2013.06.004

[mol212025-bib-0035] Soon P and Kiaris H (2013) MicroRNAs in the tumour microenvironment: big role for small players. Endocr Relat Cancer 20, R257–R267. 23878074 10.1530/ERC-13-0119

[mol212025-bib-0036] Squadrito ML , Etzrodt M , De Palma M and Pittet MJ (2013) MicroRNA‐mediated control of macrophages and its implications for cancer. Trends Immunol 34, 350–359. 23498847 10.1016/j.it.2013.02.003 PMC3700601

[mol212025-bib-0037] Stahlberg A , Rusnakova V and Kubista M (2013) The added value of single‐cell gene expression profiling. Brief Funct Genomics 12, 81–89. 23393397 10.1093/bfgp/elt001

[mol212025-bib-0038] Swartz MA , Iida N , Roberts EW , Sangaletti S , Wong MH , Yull FE , Coussens LM and DeClerck YA (2012) Tumor microenvironment complexity: emerging roles in cancer therapy. Cancer Res 72, 2473–2480. 22414581 10.1158/0008-5472.CAN-12-0122 PMC3653596

[mol212025-bib-0039] Tahiri A , Leivonen SK , Luders T , Steinfeld I , Ragle Aure M , Geisler J , Makela R , Nord S , Riis ML , Yakhini Z *et al* (2014) Deregulation of cancer‐related miRNAs is a common event in both benign and malignant human breast tumors. Carcinogenesis 35, 76–85. 24104550 10.1093/carcin/bgt333

[mol212025-bib-0040] Tjomsland V , Niklasson L , Sandstrom P , Borch K , Druid H , Bratthall C , Messmer D , Larsson M and Spangeus A (2011) The desmoplastic stroma plays an essential role in the accumulation and modulation of infiltrated immune cells in pancreatic adenocarcinoma. Clin Dev Immunol 2011, 212810. 22190968 10.1155/2011/212810 PMC3235447

[mol212025-bib-0041] de Visser KE , Eichten A and Coussens LM (2006) Paradoxical roles of the immune system during cancer development. Nat Rev Cancer 6, 24–37. 16397525 10.1038/nrc1782

[mol212025-bib-0042] Volinia S , Galasso M , Sana ME , Wise TF , Palatini J , Huebner K and Croce CM (2012) Breast cancer signatures for invasiveness and prognosis defined by deep sequencing of microRNA. Proc Natl Acad Sci U S A 109, 3024–3029. 22315424 10.1073/pnas.1200010109 PMC3286983

[mol212025-bib-0043] Wagner M and Wiig H (2015) Tumor interstitial fluid formation, characterization, and clinical implications. Front Oncol 5, 115. 26075182 10.3389/fonc.2015.00115 PMC4443729

[mol212025-bib-0044] Weber JA , Baxter DH , Zhang S , Huang DY , Huang KH , Lee MJ , Galas DJ and Wang K (2010) The microRNA spectrum in 12 body fluids. Clin Chem 56, 1733–1741. 20847327 10.1373/clinchem.2010.147405 PMC4846276

[mol212025-bib-0045] Wu H , Neilson JR , Kumar P , Manocha M , Shankar P , Sharp PA and Manjunath N (2007) miRNA profiling of naive, effector and memory CD8 T cells. PLoS One 2, e1020. 17925868 10.1371/journal.pone.0001020 PMC2000354

[mol212025-bib-0046] Wurtz SO , Moller S , Mouridsen H , Hertel PB , Friis E and Brunner N (2008) Plasma and serum levels of tissue inhibitor of metalloproteinases‐1 are associated with prognosis in node‐negative breast cancer: a prospective study. Mol Cell Proteomics 7, 424–430. 17998244 10.1074/mcp.M700305-MCP200

[mol212025-bib-0047] Yang M , Chen J , Su F , Yu B , Su F , Lin L , Liu Y , Huang JD and Song E (2011) Microvesicles secreted by macrophages shuttle invasion‐potentiating microRNAs into breast cancer cells. Mol Cancer 10, 117. 21939504 10.1186/1476-4598-10-117 PMC3190352

[mol212025-bib-0048] Zearo S , Kim E , Zhu Y , Zhao JT , Sidhu SB , Robinson BG and Soon P (2014) MicroRNA‐484 is more highly expressed in serum of early breast cancer patients compared to healthy volunteers. BMC Cancer 14, 200. 24641801 10.1186/1471-2407-14-200 PMC3995145

[mol212025-bib-0049] Zhang J , Li S , Li L , Li M , Guo C , Yao J and Mi S (2015) Exosome and exosomal microRNA: trafficking, sorting, and function. Genomics Proteomics Bioinformatics 13, 17–24. 25724326 10.1016/j.gpb.2015.02.001 PMC4411500

[mol212025-bib-0050] Zhou W , Shi G , Zhang Q , Wu Q , Li B and Zhang Z (2014) MicroRNA‐20b promotes cell growth of breast cancer cells partly via targeting phosphatase and tensin homologue (PTEN). Cell Biosci 4, 62. 25364498 10.1186/2045-3701-4-62 PMC4216355

[mol212025-bib-0051] Zhu D , Pan C , Li L , Bian Z , Lv Z , Shi L , Zhang J , Li D , Gu H , Zhang CY *et al* (2013) MicroRNA‐17/20a/106a modulate macrophage inflammatory responses through targeting signal‐regulatory protein alpha. J Allergy Clin Immunol 132, 426–436, e428. 23562609 10.1016/j.jaci.2013.02.005 PMC5882493

