# Supplementary figures and images for: Profiling of microRNAs in tumor interstitial fluid of breast tumors – a novel resource to identify biomarkers for prognostic classification and detection of cancer
Source: Mol Oncol. 2016 Dec 12;11(2):220–34. doi: 10.1002/1878-0261.12025 (PMC5527454; doi:10.1002/1878-0261.12025)

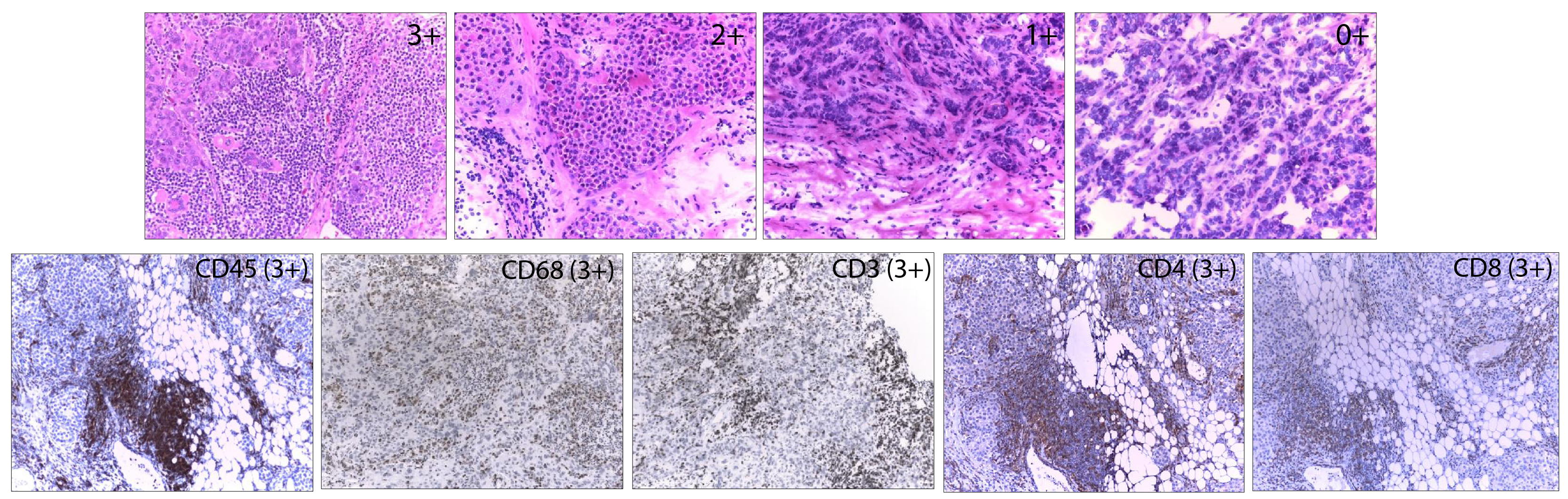

Supplement: Supplementary file 1 — Fig. S1. A representative example of the distribution of TILs that were detected in tumor biopsies based on HE and IHC staining. [file MOL2-11-220-s001.tif]

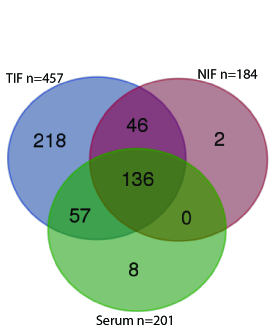

Supplement: Supplementary file 2 — Fig. S2. A Venn diagram represents the microRNAs that were detected in > 30% of the TIF (n = 457), NIF (n = 184), and serum (n = 201) samples. [file MOL2-11-220-s002.tif]

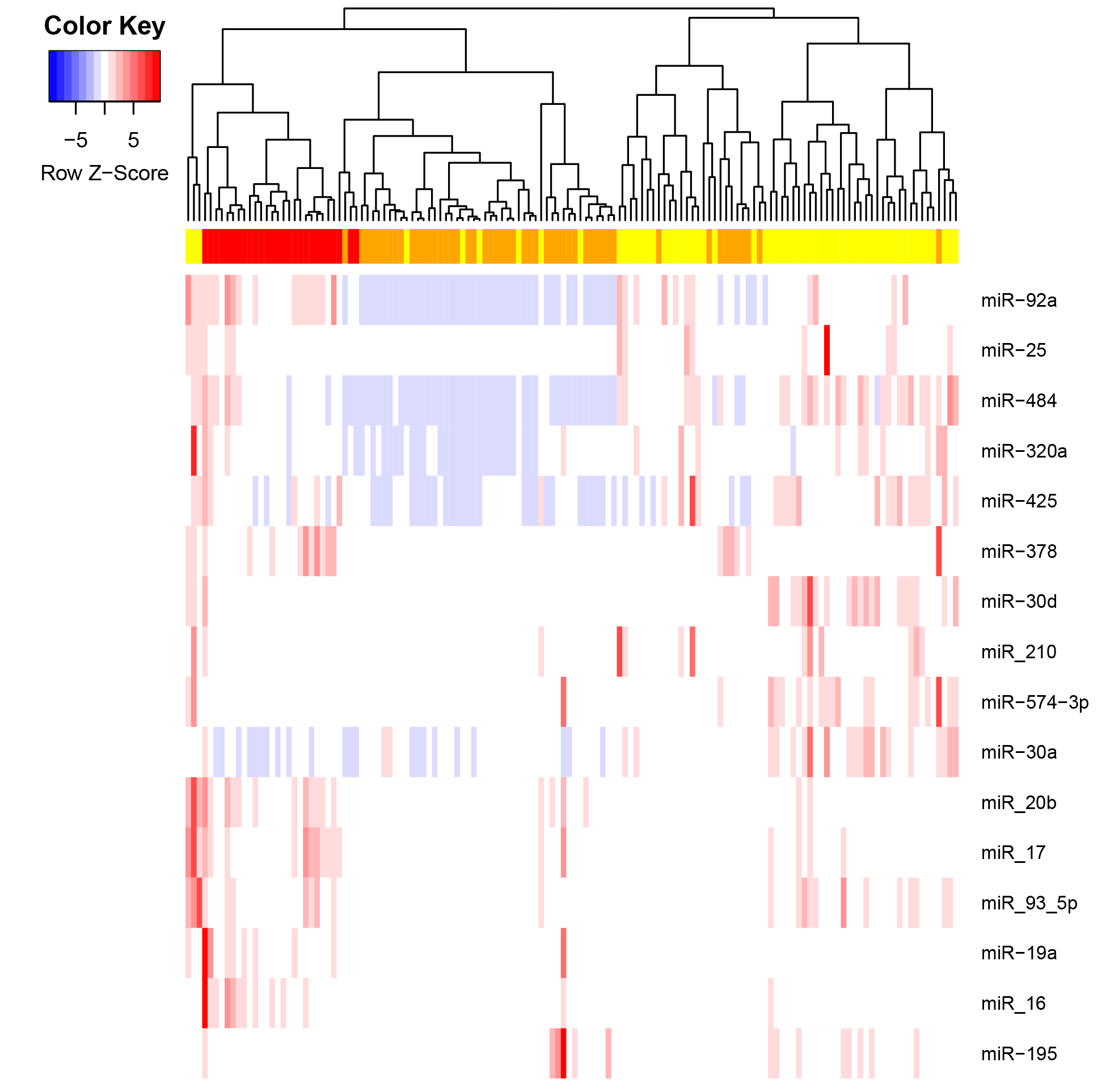

Supplement: Supplementary file 3 — Fig. S3. Hierarchical clustering of the 16 identified biomarker candidates. [file MOL2-11-220-s003.tif]

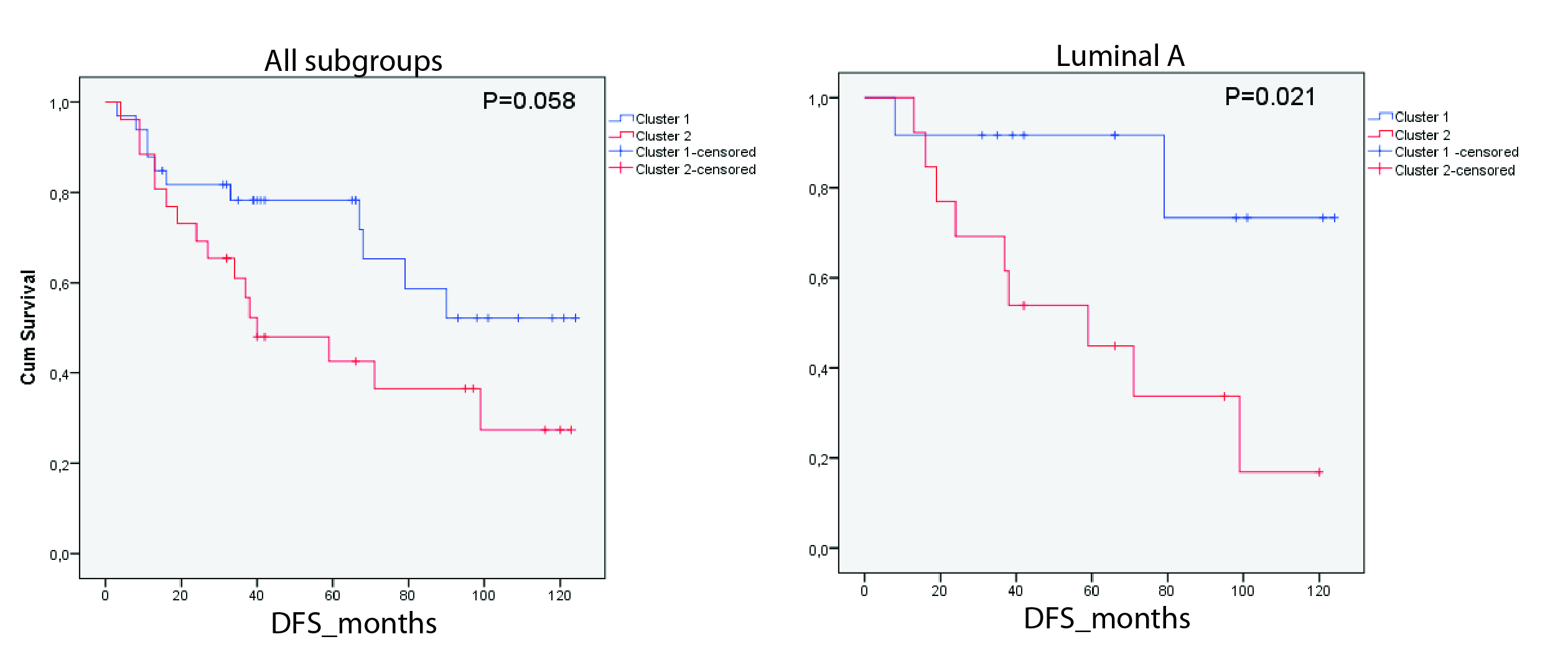

Supplement: Supplementary file 4 — Fig. S4. Kaplan–Meier survival plots and log‐rank test with P‐values for the two clusters of microRNA data. [file MOL2-11-220-s004.tif]
