# Supplementary material for: Profiling of microRNAs in tumor interstitial fluid of breast tumors – a novel resource to identify biomarkers for prognostic classification and detection of cancer
Source: Mol Oncol. 2016 Dec 12;11(2):220–34. doi: 10.1002/1878-0261.12025 (PMC5527454; doi:10.1002/1878-0261.12025)
Supplement: Supplementary file 5 — Doc. S1. Methods. [file MOL2-11-220-s005.docx]

# Supplementary Methods

Criteria for high risk cancer

The criteria for high risk cancer applied by Danish Cooperative Breast Cancer Group are age below 35 years old, and/or tumor diameter of more than 20 mm, and/or histological malignancy grade 2 or 3, and/or negative estrogen and progesterone receptor status, and/or positive axillary status. Patients received no treatment prior to surgery.

microRNA profiling

Profiling of 754 microRNAs in TIF, NIF and serum were performed using TaqMan® Low Density Arrays (TLDA, cat# 4444913, Applied Biosystem, Foster City, CA). RNA > 1000 base pairs of length were extracted from 500 µl of serum sample using miRCURY™ RNA isolation kit for biofluids (cat#300113, Exiqon, Denmark) according to manufactures protocol. Quantity and quality were determined with the NanoDrop ND-1000 spectrophotometer (Thermo Scientific, Wilmington, DE). The amount of TIF and NIF samples used for RNA isolation was based on the protein concentrations, to ensure equal loading of material. First, the samples were reverse transcribed using megaplex™ RT primer pool (pool A and B, cat#4444745, Applied Biosystem, Foster City, CA) and TaqMan® MicroRNA reverse transcription kit (cat#4366596, Applied Biosystem, Foster City, CA), with 30 ng RNA as input. All the cDNA was preamplified (pool A and B, cat#4444748, TaqMan® preamp master mix, cat#4391128, Applied Biosystem, Foster City, CA) before they were applied to the TLDA cards, containing 754 unique primers, and loaded onto the 7900HT Fast thermocycler system (Applied Biosystem, Foster City, CA) for analysis. The protocol from supplier (PN 4399721) was followed for all the procedures.

Tumor samples (n=54) and corresponding TIFs isolated from some of these tumor samples (n=14) were analyzed on high density hybridization arrays with 2549 human miRNAs represented, based on miRBase database release 21 (Human microRNA Microarrays, 8x60K, v.21, G4872A, Agilent Technologies, Santa Clara, CA). 100 ng of totRNAs from TIF and tumor samples were added the reaction. One cyanine 3-pCp molecule was ligated to the 3’ end of an RNA molecule and the labeled RNAs were hybridized on arrays at 55°C for 20 hours. The arrays were scanned using an Agilent Microarray scanner, and the raw data preprocessed using Agilent’s Feature Extraction Software v.10.7.3.1. The protocol from the supplier was followed for all the steps in the procedure (G4170-90011 v.3.1.1). Total RNA extraction from tumor tissue was performed using standard TriZol methods (Invitrogen, Carlsbad, CA), specified by the manufacturer’s instruction.

Data normalization

For data generated from the TLDA cards, microRNAs that were present in less than 30% of all the samples were excluded. All of the values were log2-transformed prior to statistical analysis. When analyzing body fluids, non-determined values were most likely due to biological issues and not technical issues, and thus, they were replaced with the lowest value detected on a linear scale. For data generated from the hybridization arrays, microRNAs with a standard deviation (SD) value < 1 were excluded, resulting in 1060 microRNAs available for further analysis. Correlation analysis was performed using 730 microRNAs present on both TLDA cards and hybridization arrays.

Statistical analysis

The distribution of microRNA expression across the samples was skewed due to the high proportion of low abundant microRNAs. Thus, non-parametric statistical tests were used. Venn diagrams were created at: <http://bioinformatics.psb.ugent.be/cgibin/liste/Venn/calculate_venn.htpl>. For pathway analysis, QIAGEN’s Ingenuity® Pathway Analysis (QIAGEN, Redwood City, [www.qiagen.com/ingenuity](http://www.qiagen.com/ingenuity)) was utilized. Only experimentally observed targets of microRNAs were used to identify potentially altered molecular canonical pathways. A FDR < 0.05 was considered significant. Pathways and molecular interactions were generated with the use of QIAGEN’s Ingenuity® iReport (QIAGEN, Redwood City, [www.qiagen.com/ingenuity](http://www.qiagen.com/ingenuity)).

Detection of 61 unique microRNAs in TIF samples: candidate breast cancer biomarkers in serum

To identify tumor-associated biomarkers in serum, we selected microRNAs present in high abundance in TIF samples relative to NIF samples and with measurable levels in serum. The Wilcoxon Rank test identified 266 microRNAs with a significantly higher abundance in TIF samples relative to NIF samples (FDR < 0.01, Supplementary Table 4). Of these, 61 microRNAs were detected in more than 75% of the serum samples. It is not expected that a marker can be detected in 100 % of the samples, but a robust marker should be measurable in the majority of the samples. In a meta-study of circulating microRNAs in breast cancer, the overall sensitivity of microRNAs was 82 %. We chose to put the threshold for detection on 75 % (Cui et al, 2015).
