# Supplementary material for: Profiling of microRNAs in tumor interstitial fluid of breast tumors – a novel resource to identify biomarkers for prognostic classification and detection of cancer
Source: Mol Oncol. 2016 Dec 12;11(2):220–34. doi: 10.1002/1878-0261.12025 (PMC5527454; doi:10.1002/1878-0261.12025)
Supplement: Supplementary file 6 — Table S1. An average expression of Ki67 used for subtype estimation and the cutoff of KI67 positivity was assigned in accordance with the currently accepted criteria (Esposito et al., 2015) and intrinsic subtypes were assigned as shown in the table, where the luminal B subtype was divided in two according to HER2‐status. Table S2. An overview of the samples included in the microRNA profiling. Table S3. Antibodies used in this study. Table S4. Spearman Rank Correlation identified microRNAs correlated between TIF, NIF, Serum and tumor. P < 0.05 is regarded as significant. Table S5. Wilcoxon Rank test identified 266 microRNAs with significantly higher abundance in TIF relatively to NIF (FDR < 0.01). Table S6. 61 microRNAs were identified using the criteria: Up in TIF vs NIF (FDR < 0.01) and expressed in more than 75% of serum samples. Table S7. The difference in abundance of the 61 candidate microRNAs were tested using student's t‐test and 52 microRNAs showed significantly higher abundance in tumor mass vs TIF. Table S8. MicroRNA profiling in serum of Chinese breast cancer patients. Table S9. Out of the 457 microRNA in TIF, the presence of TILs and tumor percentage contributed significantly to the variation of 179 microRNAs (FDR < 0.05). Table S10. Pathway analyses were performed for microRNAs significantly correlated with subgroups of TILs and adipocytes. Table S11. Kruskal‐Wallis Anova test was performed to identify microRNAs with differential distribution between the subgroups. [file MOL2-11-220-s006.docx]

**SUPPLEMENTARY TABLES**

Supplementary Table 1: An average expression of Ki67 used for subtype estimation and the cutoff of KI67 positivity was assigned in accordance with the currently accepted criteria (Esposito et al., 2015) and intrinsic subtypes were assigned as shown in the table, where the luminal B subtype was divided in two according to HER2-status

| **Intrinsic subtype** | **ER** | **HER2** | **Ki67average** |
| --- | --- | --- | --- |
| Luminal A | + | - | <15% |
| Luminal B | + | - | ≥15% |
| Luminal B HER2+ | + | + | Any |
| TNBC | - | - | Any |
| HER2+ | - | HER + | Any |

Supplementary Table 2: An overview of the samples included in the microRNA profiling. Clinical information like subtype, size, grade, Her2 expression and receptor status are included.

| ID_TIF | ID_Tumor | ID_NIF | ID_serum | Subtype | Size | Gr | Her2 (IHC) | ER | PgR |
| --- | --- | --- | --- | --- | --- | --- | --- | --- | --- |
| 1 | 1 |  |  | Her2 | III | 2 | 3+ | ER- | PGR- |
| 2 | 2 |  | 2 | LumA | III | 2 | 0 | ER+ | PGR+ |
| 3 | 3 |  |  | Her2 | II | 3 | 3+ | ER- | PGR- |
| 4 |  |  | 4 | Her2 | I | 3 | 3+ | ER- | PGR- |
| 5 | 5 | 5 |  | LumA | II | 1 | 1+ | ER+ | PGR+ |
| 6 | 6 | 6 |  | LumA | II | 2 | 0 | ER+ | PGR+ |
| 7 | 7 | 7 |  | LumB | II | 3 | 2+ | ER+ | PGR+ |
| 8 |  | 8 | 8 | TNBC | II | 3 | 1+ | ER- | PGR- |
| 9 | 9 | 9 |  | LumA | II | 2 | 2+ | ER+ | PGR- |
| 10 | 10 | 10 |  | TNBC | II | 3 | 0 | ER- | PGR- |
| 11 | 11 | 11 |  | TNBC | II | 1 | 1+ | ER- | PGR- |
| 12 | 12 | 12 |  | LumA | III | 2 | 1+ | ER+ | PGR+ |
| 13 |  | 13 |  | LumA | III | 2 | 2+ | ER+ | PGR+ |
| 14 | 14 | 14 | 14 | LumB | I | 2 | 0 | ER+ | PGR+ |
| 15 | 15 | 15 |  | LumA | IV | 2 | 1+ | ER+ | PGR+ |
| 16 | 16 | 16 | 16 | Her2 | II | 3 | 3+ | ER- | PGR- |
| 17 | 17 |  | 17 | Her2 | II | 3 | 3+ | ER- | PGR- |
| 18 | 18 | 18 |  | LumA | III | 2 | 0 | ER+ | PGR- |
| 19 | 19 | 19 |  | LumA | III | 1 | 1+ | ER+ | PGR+ |
| 20 | 20 | 20 |  | LumA | III | 2 | 0 | ER+ | PGR+ |
| 21 | 21 | 21 |  | LumA | II | 2 | 0 | ER+ | PGR+ |
| 22 | 22 | 22 |  | LumA | II | 1 | 0 | ER+ | PGR+ |
| 23 | 23 | 23 |  | Her2 | II | 2 | 3+ | ER- | PGR- |
| 24 | 24 | 24 |  | Her2 | I | 3 | 3+ | ER- | PGR- |
| 25 | 25 | 25 |  | LumA | II | 2 | 1+ | ER+ | PGR+ |
| 26 | 26 | 26 |  | LumB | III | 3 | 2+ | ER+ | PGR- |
| 27 | 27 | 27 | 27 | LumA | II | 3 | 1+ | ER+ | PGR+ |
| 28 | 28 | 28 |  | LumA | II | 2 | 1+ | ER+ | PGR+ |
| 29 | 29 | 29 |  | TNBC | I | 3 | 0 | ER- | PGR- |
| 30 | 30 |  | 30 | LumA | II | 2 | 0 | ER+ | PGR+ |
| 31 | 31 | 31 |  | LumB | II | 3 | 2+ | ER+ | PGR+ |
| 32 | 32 | 32 | 32 | LumB | II | 3 | 1+ | ER+ | PGR+ |
| 33 | 33 | 33 | 33 | LumA | III | 2 | 1+ | ER+ | PGR- |
| 34 | 34 | 34 | 34 | LumA | III | 2 | 1+ | ER+ | PGR+ |
| 35 | 35 | 35 | 35 | LumA | III | 2 | 0 | ER+ | PGR+ |
| 36 | 36 | 36 | 36 | LumA | I | 2 | 1+ | ER+ | PGR+ |
| 37 | 37 | 37 | 37 | TNBC | II | 3 | 0 | ER- | PGR- |
| 38 | 38 |  |  | TNBC | II | 3 | 0 | ER- | PGR- |
| 39 | 39 | 39 | 39 | LumA | I | 2 | 0 | ER+ | PGR- |
| 40 | 40 | 40 |  | TNBC | II | 3 | 1+ | ER+ | PGR- |
| 41 | 41 | 41 | 41 | LumA | I | 3 | 2+ | ER+ | PGR+ |
| 42 | 42 | 42 | 42 | LumB | II | 2 | 0 | ER+ | PGR+ |
| 43 | 43 |  | 43 | LumB | II | 3 | 0 | ER+ | PGR+ |
| 44 | 44 | 44 | 44 | Her2 | II | 3 | 3+ | ER- | PGR- |
| 45 | 45 |  | 45 | TNBC | III | 3 | 1+ | ER- | PGR- |
| 46 | 46 | 46 |  | LumB | II | 3 | 2+ | ER+ | PGR+ |
| 47 | 47 | 47 |  | LumB | II | 3 | 0 | ER+ | PGR- |
| 48 | 48 | 48 |  | TNBC | II | 3 | 2+ | ER- | PGR- |
| 49 | 49 | 49 | 49 | LumA | II | 2 | 2+ | ER+ | ND |
| 50 | 50 | 50 | 50 | TNBC | II | 3 | 1+ | ER- | PGR- |
| 51 | 51 | 51 | 51 | LumA | II | 3 | 1+ | ER+ | PGR- |
| 52 | 52 | 52 | 52 | LumB | II | 3 | 3+ | ER+ | PGR- |
| 53 |  | 53 |  | LumB | IV | 2 | 1+ | ER+ | PGR+ |
| 54 | 54 | 54 |  | TNBC | IV |  | 2+ | ER- | PGR- |
| 55 | 55 | 55 |  | LumB | II | 2 | 2+ | ER+ | PGR+ |
| 56 | 56 | 56 | 56 | LumB | II | 3 | 2+ | ER+ | PGR+ |
| 57 |  | 57 |  | LumB | II | 3 | 2+ | ER+ | ND |
| 58 | 58 | 58 | 58 | LumA | II | 3 | 2+ | ER+ | PGR+ |
| 59 | 59 | 59 |  | TNBC | III |  | 2+ | ER- | ND |
| 60 |  | 60 | 60 | TNBC | III | 3 | 1+ | ER- | PGR- |

Supplementary Table 3**:** Antibodies used in this study

| **Marker** | **Antibody** | **Dilution used** | **Vendor** |
| --- | --- | --- | --- |
| **CK19** | Monoclonal mouse (clone 4E8) | 1:1000 | ThermoFischer Scientific |
| **Ki67** | Monoclonal mouse (clone MIB-1) | 1:200 | DAKO |
| **ER** | Monoclonal mouse (clone 1D5) | 1:200 | DAKO |
| **PgR** | Monoclonal mouse (synthetic peptide directed towards the N-terminal end) | 1:200 | DAKO |
| **HER2** | Polyclonal rabbit (HercepTest) | 1:300 | DAKO |
| **CD3** | Polyclonal rabbit (synthetic peptide from the intracellular part of the ε-chain of human CD3) | 1:200 | DAKO |
| **CD4** | Monoclonal mouse (clone IS 649) | 1:25 | DAKO |
| **CD8** | Monoclonal Mouse (clone 144B) | 1:100 | DAKO |
| **CD45** | Monoclonal mouse (clone 2B11+PD7/26) | 1:400 | DAKO |
| **CD68** | Monoclonal mouse (clone PG-M1) | 1:100 | DAKO |

Supplementary Table 4: Spearman Rank Correlation identified microRNAs correlated between TIF, NIF, Serum and tumor. P<0.05 is regarded as significant.

| Correlated mir TIF vs tumor | |  | Correlated mir TIF vs NIF | |  | Correlated mir TIF vs Serum | |  | Correlated mir TIF vs Serum | |
| --- | --- | --- | --- | --- | --- | --- | --- | --- | --- | --- |
| miRNA (n=62) | **p-value** |  | **miRNA (n=61)** | **p-value** |  | **miRNA (n=48)** | **p-value** |  | **miRNA (n=39)** | **p-value** |
| hsa-miR-375 | 8.50E-09 |  | hsa-miR-657 | 9.50E-07 |  | hsa-miR-220c | 2.20E-05 |  | hsa-miR-18a-3p | 3.50E-150 |
| hsa-miR-205-5p | 1.00E-08 |  | hsa-miR-141-5p | 1.00E-05 |  | hsa-miR-519e-5p | 1.50E-04 |  | hsa-miR-323a-3p | 1.60E-04 |
| hsa-miR-127-3p | 1.20E-06 |  | hsa-miR-519e-5p | 1.50E-05 |  | hsa-miR-105-3p | 9.50E-04 |  | hsa-miR-32-3p | 2.00E-04 |
| hsa-miR-30c-2-3p | 3.40E-06 |  | hsa-miR-1272 | 4.00E-05 |  | hsa-miR-513c-5p | 9.50E-04 |  | hsa-miR-1265 | 1.40E-03 |
| hsa-miR-30a-3p | 4.00E-06 |  | hsa-miR-1290 | 5.60E-05 |  | hsa-miR-920 | 9.50E-04 |  | hsa-miR-216b-5p | 1.40E-03 |
| hsa-miR-342-5p | 6.40E-06 |  | hsa-miR-1203 | 2.30E-04 |  | hsa-miR-199a-3p | 6.00E-03 |  | hsa-miR-519e-5p | 1.40E-03 |
| hsa-miR-142-5p | 1.00E-05 |  | hsa-miR-551b-5p | 2.30E-04 |  | hsa-miR-561-3p | 9.90E-03 |  | hsa-miR-659-3p | 1.40E-03 |
| hsa-miR-30a-5p | 1.80E-04 |  | hsa-miR-563 | 7.00E-04 |  | hsa-miR-331-3p | 1.10E-02 |  | hsa-miR-380-3p | 3.60E-03 |
| hsa-miR-224-5p | 2.30E-04 |  | hsa-miR-1233-3p | 1.50E-03 |  | hsa-miR-623 | 1.20E-02 |  | hsa-miR-432-3p | 3.60E-03 |
| hsa-miR-203a-3p | 2.90E-04 |  | hsa-miR-520c-3p | 2.00E-03 |  | hsa-miR-126-3p | 1.20E-02 |  | hsa-miR-505-3p | 3.60E-03 |
| hsa-miR-7-5p | 3.30E-04 |  | hsa-miR-30d-3p | 2.60E-03 |  | hsa-miR-340-5p | 1.20E-02 |  | hsa-miR-130a-3p | 9.60E-03 |
| hsa-miR-425-5p | 3.30E-04 |  | hsa-miR-579-3p | 2.70E-03 |  | hsa-miR-30a-5p | 1.20E-02 |  | hsa-miR-15a-3p | 1.00E-02 |
| hsa-miR-141-3p | 5.30E-04 |  | hsa-miR-937-3p | 3.50E-03 |  | hsa-miR-132-3p | 1.30E-02 |  | hsa-miR-19b-3p | 1.50E-02 |
| hsa-miR-423-5p | 5.80E-04 |  | hsa-miR-345-5p | 3.60E-03 |  | hsa-miR-581 | 1.50E-02 |  | hsa-miR-93-5p | 1.90E-02 |
| hsa-miR-429 | 9.90E-04 |  | hsa-miR-526b-5p | 4.10E-03 |  | hsa-miR-15b-5p | 1.50E-02 |  | hsa-miR-181a-5p | 2.00E-02 |
| hsa-miR-145-5p | 1.40E-03 |  | hsa-miR-944 | 5.20E-03 |  | hsa-miR-495-3p | 1.50E-02 |  | hsa-miR-362-3p | 2.10E-02 |
| hsa-miR-342-3p | 1.40E-03 |  | hsa-miR-125b-5p | 5.30E-03 |  | hsa-miR-222 | 1.50E-02 |  | hsa-miR-485-5p | 2.10E-02 |
| hsa-miR-330-3p | 1.40E-03 |  | hsa-miR-661 | 5.60E-03 |  | hsa-miR-320B | 1.60E-02 |  | hsa-miR-142-5p | 2.30E-02 |
| hsa-miR-200a-3p | 2.70E-03 |  | hsa-miR-92a-1-5p | 5.60E-03 |  | hsa-miR-873-5p | 1.60E-02 |  | hsa-miR-337-3p | 2.30E-02 |
| hsa-miR-320b | 3.90E-03 |  | hsa-miR-1276 | 7.80E-03 |  | hsa-miR-28-5p | 1.90E-02 |  | hsa-miR-515-5p | 2.30E-02 |
| hsa-miR-424-5p | 5.70E-03 |  | hsa-miR-518c-5p | 8.60E-03 |  | hsa-miR-1250-5p | 2.00E-02 |  | hsa-miR-485-3p | 2.50E-02 |
| hsa-miR-515-5p | 5.70E-03 |  | hsa-miR-218-2-3p | 8.70E-03 |  | hsa-miR-337-3p | 2.00E-02 |  | hsa-miR-181a-2-3p | 2.60E-02 |
| hsa-miR-33a-5p | 7.10E-03 |  | hsa-miR-875-5p | 9.70E-03 |  | hsa-miR-891b | 2.00E-02 |  | hsa-miR-219a-1-3p | 2.60E-02 |
| hsa-miR-320a | 7.70E-03 |  | hsa-miR-758-3p | 1.10E-02 |  | hsa-miR-548m | 2.10E-02 |  | hsa-miR-525-5p | 2.60E-02 |
| hsa-miR-517-5p | 8.70E-03 |  | hsa-miR-331-3p | 1.10E-02 |  | hsa-miR-410-3p | 2.10E-02 |  | hsa-miR-556-3p | 2.60E-02 |
| hsa-miR-19b-3p | 9.40E-03 |  | hsa.miR.1274B | 1.20E-02 |  | hsa-miR-520a-3p | 2.30E-02 |  | hsa-miR-379-5p | 2.60E-02 |
| hsa-miR-19a-3p | 1.10E-02 |  | hsa-miR-124-5p | 1.30E-02 |  | hsa-miR-330-3p | 2.30E-02 |  | hsa-miR-1254 | 2.60E-02 |
| hsa-miR-17-3p | 1.10E-02 |  | hsa-miR-320B | 1.30E-02 |  | hsa-miR-20a-5p | 2.50E-02 |  | hsa-miR-191-3p | 2.60E-02 |
| hsa-miR-193b-5p | 1.30E-02 |  | hsa-miR-181a-3p | 1.40E-02 |  | hsa-miR-1276 | 2.60E-02 |  | hsa-miR-767-5p | 2.60E-02 |
| hsa-miR-99a-5p | 1.30E-02 |  | hsa-miR-16-1-3p | 1.50E-02 |  | hsa-miR-30a-3p | 2.80E-02 |  | hsa-miR-652-3p | 2.80E-02 |
| hsa-miR-21-3p | 1.30E-02 |  | hsa-miR-26a-1-3p | 1.70E-02 |  | hsa-miR-1825 | 2.90E-02 |  | hsa-miR-1298-5p | 3.30E-02 |
| hsa-miR-30e-3p | 1.40E-02 |  | hsa-miR-483-3p | 1.80E-02 |  | hsa-miR-106b-5p | 2.90E-02 |  | hsa-miR-106b-5p | 3.90E-02 |
| hsa-miR-197-3p | 1.40E-02 |  | hsa-miR-582-5p | 2.00E-02 |  | hsa-miR-342-3p | 3.00E-02 |  | hsa-miR-186-5p | 4.20E-02 |
| hsa-miR-92a-3p | 1.40E-02 |  | hsa-miR-656-3p | 2.10E-02 |  | hsa-miR-19b-3p | 3.10E-02 |  | hsa-miR-502-3p | 4.30E-02 |
| hsa-miR-1291 | 1.70E-02 |  | hsa-miR-541-3p | 2.10E-02 |  | hsa-miR-98-5p | 3.10E-02 |  | hsa-miR-520a-3p | 4.30E-02 |
| hsa-miR-370-3p | 1.70E-02 |  | hsa-miR-1180-3p | 2.20E-02 |  | hsa-miR-660-5p | 3.10E-02 |  | hsa-miR-1303 | 4.30E-02 |
| hsa-miR-501-5p | 1.70E-02 |  | hsa-miR-616-3p | 2.20E-02 |  | hsa-miR-1227-3p | 3.50E-02 |  | hsa-miR-532-5p | 4.50E-02 |
| hsa-miR-605-5p | 1.90E-02 |  | hsa-miR-600 | 2.40E-02 |  | hsa-miR-744-5p | 3.50E-02 |  | hsa-miR-17-5p | 4.60E-02 |
| hsa-miR-25-3p | 1.90E-02 |  | hsa-miR-151a-3p | 2.50E-02 |  | hsa-miR-345-5p | 3.90E-02 |  | hsa-miR-340-3p | 4.80E-02 |
| hsa-miR-376a-3p | 1.90E-02 |  | hsa-miR-543 | 2.50E-02 |  | hsa-miR-151a-5P | 3.90E-02 |  |  |  |
| hsa-let-7b-5p | 1.90E-02 |  | hsa-miR-99b-3p | 2.80E-02 |  | hsa-miR-19a-3p | 3.90E-02 |  |  |  |
| hsa-miR-200b-3p | 2.00E-02 |  | hsa-miR-425-3p | 3.00E-02 |  | hsa-miR-1249-3p | 4.00E-02 |  |  |  |
| hsa-miR-210-3p | 2.00E-02 |  | hsa-miR-451a | 3.10E-02 |  | hsa-miR-30c-5p | 4.00E-02 |  |  |  |
| hsa-miR-27a-3p | 2.00E-02 |  | hsa-miR-518a-3p | 3.20E-02 |  | hsa-miR-33a-3p | 4.50E-02 |  |  |  |
| hsa-miR-335-3p | 2.20E-02 |  | hsa-miR-886-3p | 3.30E-02 |  | hsa-miR-30b-5p | 4.80E-02 |  |  |  |
| hsa-miR-150-5p | 2.20E-02 |  | hsa-miR-609 | 3.60E-02 |  | hsa-miR-362-5p | 4.90E-02 |  |  |  |
| hsa-miR-410-3p | 2.40E-02 |  | hsa-miR-617 | 3.80E-02 |  | hsa-miR-376c-3p | 4.90E-02 |  |  |  |
| hsa-miR-142-3p | 2.50E-02 |  | hsa-miR-449b-5p | 3.80E-02 |  | hsa-miR-892b | 5.00E-02 |  |  |  |
| hsa-miR-191-5p | 2.60E-02 |  | hsa-miR-644a | 3.90E-02 |  |  |  |  |  |  |
| hsa-miR-149-5p | 2.60E-02 |  | hsa-miR-1265 | 4.00E-02 |  |  |  |  |  |  |
| hsa-miR-181a-3p | 3.20E-02 |  | hsa-miR-410-3p | 4.00E-02 |  |  |  |  |  |  |
| hsa-miR-539-5p | 3.30E-02 |  | hsa-miR-1184 | 4.10E-02 |  |  |  |  |  |  |
| hsa-miR-146a-5p | 3.30E-02 |  | hsa.miR.1274A | 4.20E-02 |  |  |  |  |  |  |
| hsa-miR-99b-5p | 3.50E-02 |  | hsa-miR-615-3p | 4.20E-02 |  |  |  |  |  |  |
| hsa-miR-93-5p | 3.60E-02 |  | hsa-miR-720 | 4.20E-02 |  |  |  |  |  |  |
| hsa-miR-590-5p | 3.60E-02 |  | hsa-miR-545-3p | 4.30E-02 |  |  |  |  |  |  |
| hsa-miR-939-5p | 4.20E-02 |  | hsa-miR-22-3p | 4.50E-02 |  |  |  |  |  |  |
| hsa-miR-181c-5p | 4.30E-02 |  | hsa-let-7c* | 4.60E-02 |  |  |  |  |  |  |
| hsa-miR-339-3p | 4.30E-02 |  | hsa-miR-100-3p | 4.60E-02 |  |  |  |  |  |  |
| hsa-miR-643 | 4.40E-02 |  | hsa-miR-92a-2-5p | 4.90E-02 |  |  |  |  |  |  |
| hsa-miR-200c-3p | 4.40E-02 |  | hsa-miR-20a-3p | 4.90E-02 |  |  |  |  |  |  |
| hsa-miR-152-3p | 4.70E-02 |  |  |  |  |  |  |  |  |  |

Supplementary Table 5: Wilcoxon Rank test identified 266 microRNAs with significantly higher abundance in TIF relatively to NIF (FDR<0.01)

| microRNA | p-value | FDR | microRNA | p-value | FDR | microRNA | p-value | FDR |
| --- | --- | --- | --- | --- | --- | --- | --- | --- |
| hsa-let-7a-5p | 5.69E-09 | 1.46E-08 | hsa-miR-199a-3p | 7.14E-06 | 1.18E-05 | hsa-miR-410-3p | 9.58E-05 | 0.0001 |
| hsa-let-7b-5p | 0.0006 | 0.0008 | hsa-miR-199a-5p | 5.03E-09 | 1.31E-08 | hsa-miR-423-5p | 1.09E-13 | 1.20E-12 |
| hsa-let-7d-5p | 3.75E-10 | 1.21E-09 | hsa-miR-19a-3p | 0.0036 | 0.0049 | hsa-miR-424-3p | 7.77E-07 | 1.39E-06 |
| hsa-let-7e-5p | 3.99E-09 | 1.07E-08 | hsa-miR-19b-1-5p | 4.48E-05 | 7.11E-05 | hsa-miR-424-5p | 1.26E-08 | 3.01E-08 |
| hsa-let-7f-5p | 7.62E-08 | 1.58E-07 | hsa-miR-19b-3p | 0.0003 | 0.0004 | hsa-miR-425-5p | 1.30E-11 | 6.12E-11 |
| hsa-let-7g-5p | 3.22E-11 | 1.32E-10 | hsa-miR-200a-3p | 1.12E-13 | 1.20E-12 | hsa-miR-429 | 4.57E-09 | 1.20E-08 |
| hsa-let-7i-3p | 9.44E-08 | 1.92E-07 | hsa-miR-200b-3p | 1.99E-10 | 6.62E-10 | hsa-miR-454-3p | 8.89E-10 | 2.68E-09 |
| hsa-miR-100-5p | 8.09E-05 | 0.0001 | hsa-miR-200c-3p | 7.23E-16 | 3.23E-14 | hsa-miR-455-5p | 3.80E-09 | 1.03E-08 |
| hsa-miR-101-3p | 0.0040 | 0.0053 | hsa-miR-202-3p | 5.27E-10 | 1.69E-09 | hsa-miR-483-3p | 3.36E-07 | 6.26E-07 |
| hsa-miR-106a-5p | 0.0001 | 0.0002 | hsa-miR-203a-3p | 1.29E-07 | 2.55E-07 | hsa-miR-484 | 5.04E-15 | 1.33E-13 |
| hsa-miR-106b-3p | 1.24E-12 | 8.34E-12 | hsa-miR-205-5p | 7.67E-13 | 5.85E-12 | hsa-miR-485-5p | 1.73E-08 | 4.01E-08 |
| hsa-miR-106b-5p | 2.02E-11 | 8.99E-11 | hsa-miR-206 | 2.20E-08 | 4.93E-08 | hsa-miR-487a-3p | 3.19E-15 | 1.10E-13 |
| hsa-miR-10a-5p | 7.89E-09 | 1.95E-08 | hsa-miR-20b-5p | 0.0041 | 0.0054 | hsa-miR-487b-3p | 6.67E-07 | 1.21E-06 |
| hsa-miR-10b-3p | 0.0007 | 0.0010 | hsa-miR-210-3p | 1.71E-14 | 3.08E-13 | hsa-miR-491-5p | 3.95E-14 | 5.54E-13 |
| hsa-miR-10b-5p | 6.82E-09 | 1.72E-08 | hsa-miR-212-3p | 1.68E-09 | 4.75E-09 | hsa-miR-494-3p | 1.75E-12 | 1.12E-11 |
| hsa-miR-1180-3p | 2.53E-10 | 8.25E-10 | hsa-miR-21-3p | 3.63E-17 | 6.22E-15 | hsa-miR-495-3p | 0.0002 | 0.0004 |
| hsa-miR-1183 | 9.88E-14 | 1.17E-12 | hsa-miR-214-3p | 1.72E-11 | 7.87E-11 | hsa-miR-500a-5p | 6.90E-11 | 2.57E-10 |
| hsa-miR-1226-5p | 5.44E-13 | 4.67E-12 | hsa-miR-214-5p | 5.22E-11 | 2.08E-10 | hsa-miR-501-5p | 7.76E-09 | 1.93E-08 |
| hsa-miR-1227-3p | 1.45E-08 | 3.38E-08 | hsa-miR-21-5p | 9.53E-13 | 6.81E-12 | hsa-miR-505-3p | 3.17E-11 | 1.31E-10 |
| hsa-miR-1244 | 1.23E-09 | 3.64E-09 | hsa-miR-220b | 0.0023 | 0.0032 | hsa-miR-505-5p | 2.14E-11 | 9.30E-11 |
| hsa-miR-1254 | 2.65E-08 | 5.83E-08 | hsa-miR-221-3p | 3.98E-12 | 2.32E-11 | hsa-miR-511-5p | 0.0043 | 0.0056 |
| hsa-miR-1255b-5p | 6.72E-14 | 8.54E-13 | hsa-miR-222-3p | 5.48E-11 | 2.15E-10 | hsa-miR-517-5p | 1.10E-12 | 7.57E-12 |
| hsa-miR-125a-3p | 3.42E-09 | 9.38E-09 | hsa-miR-223-3p | 3.60E-07 | 6.64E-07 | hsa-miR-518e-3p | 1.27E-11 | 6.07E-11 |
| hsa-miR-125a-5p | 3.66E-15 | 1.14E-13 | hsa-miR-223-5p | 1.08E-07 | 2.15E-07 | hsa-miR-519b-3p | 3.52E-05 | 5.62E-05 |
| hsa-miR-125b-1-3p | 1.15E-07 | 2.27E-07 | hsa-miR-22-3p | 5.57E-10 | 1.77E-09 | hsa-miR-519e-3p | 0.0008 | 0.0011 |
| hsa-miR-125b-5p | 8.28E-10 | 2.51E-09 | hsa-miR-224-5p | 0.0050 | 0.0066 | hsa-miR-520d-3P | 8.93E-07 | 1.59E-06 |
| hsa-miR-1260a | 1.32E-08 | 3.12E-08 | hsa-miR-22-5p | 3.04E-06 | 5.19E-06 | hsa-miR-532-3p | 6.51E-12 | 3.29E-11 |
| hsa-miR-126-3p | 0.0001 | 0.0002 | hsa-miR-23a-3p | 7.45E-08 | 1.56E-07 | hsa-miR-532-5p | 1.14E-08 | 2.74E-08 |
| hsa-miR-126-5p | 2.74E-07 | 5.13E-07 | hsa-miR-23a-5p | 6.11E-10 | 1.90E-09 | hsa-miR-539-5p | 0.0038 | 0.0050 |
| hsa-miR-1267 | 8.90E-11 | 3.18E-10 | hsa-miR-24-3p | 5.96E-12 | 3.16E-11 | hsa-miR-548am-5p | 4.82E-09 | 1.26E-08 |
| hsa-miR-1271-5p | 0.0013 | 0.0018 | hsa-miR-25-3p | 5.43E-16 | 3.10E-14 | hsa-miR-548b-5p | 1.10E-12 | 7.57E-12 |
| hsa-miR-127-3p | 4.05E-08 | 8.75E-08 | hsa-miR-26a-5p | 1.27E-06 | 2.22E-06 | hsa-miR-548d-5p | 6.07E-10 | 1.90E-09 |
| hsa-miR-1274A | 9.67E-15 | 1.95E-13 | hsa-miR-26b-3p | 1.26E-09 | 3.70E-09 | hsa-miR-551b-5p | 0.0013 | 0.0018 |
| hsa-miR-1274B | 1.94E-14 | 3.32E-13 | hsa-miR-26b-5p | 2.94E-05 | 4.72E-05 | hsa-miR-564 | 8.82E-13 | 6.58E-12 |
| hsa-miR-1275 | 3.73E-16 | 2.56E-14 | hsa-miR-27a-3p | 2.72E-09 | 7.58E-09 | hsa-miR-566 | 1.26E-14 | 2.40E-13 |
| hsa-miR-1276 | 2.64E-11 | 1.12E-10 | hsa-miR-27a-5p | 8.96E-09 | 2.19E-08 | hsa-miR-574-3p | 1.27E-09 | 3.70E-09 |
| hsa-miR-1282 | 2.74E-05 | 4.41E-05 | hsa-miR-27b-3p | 2.03E-08 | 4.62E-08 | hsa-miR-576-3p | 8.20E-11 | 3.02E-10 |
| hsa-miR-128-3p | 1.07E-13 | 1.20E-12 | hsa-miR-28-3p | 7.17E-13 | 5.59E-12 | hsa-miR-579-3p | 6.44E-13 | 5.26E-12 |
| hsa-miR-1285-3p | 1.68E-16 | 1.49E-14 | hsa-miR-28-5p | 3.78E-09 | 1.03E-08 | hsa-miR-587 | 5.67E-08 | 1.19E-07 |
| hsa-miR-1290 | 1.55E-09 | 4.44E-09 | hsa-miR-296-5p | 6.11E-13 | 5.11E-12 | hsa-miR-590-3P | 1.38E-07 | 2.70E-07 |
| hsa-miR-1291 | 1.77E-13 | 1.84E-12 | hsa-miR-29a-3p | 2.78E-06 | 4.77E-06 | hsa-miR-590-5p | 4.90E-06 | 8.16E-06 |
| hsa-miR-1300 | 7.10E-13 | 5.59E-12 | hsa-miR-29b-3p | 8.66E-11 | 3.13E-10 | hsa-miR-596 | 2.31E-09 | 6.50E-09 |
| hsa-miR-1303 | 5.15E-12 | 2.80E-11 | hsa-miR-301a-5p | 1.91E-08 | 4.37E-08 | hsa-miR-601 | 0.0005 | 0.0007 |
| hsa-miR-130a-3p | 1.79E-05 | 2.93E-05 | hsa-miR-30a-3p | 3.38E-06 | 5.69E-06 | hsa-miR-622 | 7.54E-16 | 3.23E-14 |
| hsa-miR-130b-3p | 5.61E-11 | 2.16E-10 | hsa-miR-30a-5p | 9.00E-09 | 2.19E-08 | hsa-miR-623 | 1.74E-08 | 4.01E-08 |
| hsa-miR-132-3p | 0.0002 | 0.0003 | hsa-miR-30b-5p | 1.08E-11 | 5.21E-11 | hsa-miR-625-5p | 5.74E-07 | 1.05E-06 |
| hsa-miR-134-5p | 0.0006 | 0.0009 | hsa-miR-30c-5p | 5.98E-12 | 3.16E-11 | hsa-miR-628-3p | 0.0021 | 0.0028 |
| hsa-miR-139-5p | 0.0003 | 0.0004 | hsa-miR-30d-3p | 3.06E-11 | 1.28E-10 | hsa-miR-628-5p | 7.30E-05 | 0.000114388 |
| hsa-miR-140-3p | 2.03E-07 | 3.87E-07 | hsa-miR-30d-5p | 6.29E-12 | 3.27E-11 | hsa-miR-629-3p | 2.18E-05 | 3.54E-05 |
| hsa-miR-140-5p | 2.37E-05 | 3.83E-05 | hsa-miR-30e-3p | 1.22E-06 | 2.14E-06 | hsa-miR-636 | 0.0002 | 0.0002 |
| hsa-miR-141-3p | 1.44E-12 | 9.48E-12 | hsa-miR-31-3p | 0.0015 | 0.0021 | hsa-miR-638 | 5.27E-08 | 1.12E-07 |
| hsa-miR-141-5p | 0.0068 | 0.0088 | hsa-miR-31-5p | 2.32E-12 | 1.45E-11 | hsa-miR-639 | 1.55E-11 | 7.17E-11 |
| hsa-miR-142-3p | 8.12E-14 | 9.95E-13 | hsa-miR-320a | 1.40E-07 | 2.73E-07 | hsa-miR-642a-5p | 9.51E-09 | 2.30E-08 |
| hsa-miR-142-5p | 8.29E-07 | 1.48E-06 | hsa-miR-320B | 2.22E-11 | 9.54E-11 | hsa-miR-643 | 2.65E-08 | 5.83E-08 |
| hsa-miR-143-3p | 0.0047 | 0.0061 | hsa-miR-324-3p | 9.45E-13 | 6.81E-12 | hsa-miR-648 | 7.89E-08 | 1.63E-07 |
| hsa-miR-145-5p | 1.35E-09 | 3.90E-09 | hsa-miR-324-5p | 5.05E-14 | 6.67E-13 | hsa-miR-650 | 0.0007 | 0.0010 |
| hsa-miR-146a-5p | 5.11E-08 | 1.09E-07 | hsa-miR-326 | 1.62E-10 | 5.44E-10 | hsa-miR-652-3p | 1.52E-07 | 2.93E-07 |
| hsa-miR-146b-3p | 3.14E-13 | 2.84E-12 | hsa-miR-330-3p | 4.35E-08 | 9.32E-08 | hsa-miR-659-3p | 4.18E-12 | 2.39E-11 |
| hsa-miR-146b-5p | 3.07E-12 | 1.88E-11 | hsa-miR-330-5p | 0.0001 | 0.0002 | hsa-miR-660-5p | 4.05E-09 | 1.08E-08 |
| hsa-miR-148a-3p | 3.25E-06 | 5.50E-06 | hsa-miR-331-3p | 2.55E-15 | 9.71E-14 | hsa-miR-663B | 1.08E-10 | 3.77E-10 |
| hsa-miR-148b-3p | 9.32E-08 | 1.90E-07 | hsa-miR-331-5p | 3.61E-17 | 6.22E-15 | hsa-miR-671-3p | 4.04E-14 | 5.54E-13 |
| hsa-miR-149-5p | 4.94E-07 | 9.06E-07 | hsa-miR-335-3p | 1.04E-06 | 1.83E-06 | hsa-miR-708-5p | 2.02E-10 | 6.66E-10 |
| hsa-miR-150-5p | 4.14E-06 | 6.93E-06 | hsa-miR-338-3p | 6.18E-09 | 1.57E-08 | hsa-miR-7-1-3p | 1.24E-10 | 4.24E-10 |
| hsa-miR-151a-3p | 7.80E-15 | 1.78E-13 | hsa-miR-339-3p | 3.64E-13 | 3.20E-12 | hsa-miR-720 | 2.66E-14 | 4.15E-13 |
| hsa-miR-151a-5P | 7.00E-07 | 1.26E-06 | hsa-miR-339-5p | 1.07E-11 | 5.21E-11 | hsa-miR-744-3p | 2.11E-11 | 9.30E-11 |
| hsa-miR-152-3p | 0.0001 | 0.0002 | hsa-miR-33a-3p | 8.63E-11 | 3.13E-10 | hsa-miR-744-5p | 9.09E-15 | 1.95E-13 |
| hsa-miR-153-3p | 0.0030 | 0.0040 | hsa-miR-340-3p | 1.70E-07 | 3.25E-07 | hsa-miR-7-5p | 1.77E-12 | 1.12E-11 |
| hsa-miR-154-5p | 0.0036 | 0.0048 | hsa-miR-340-5p | 1.51E-07 | 2.93E-07 | hsa-miR-766-3p | 0.0030 | 0.0041 |
| hsa-miR-155-5p | 3.00E-14 | 4.47E-13 | hsa-miR-342-3p | 2.25E-13 | 2.20E-12 | hsa-miR-769-3p | 1.04E-07 | 2.08E-07 |
| hsa-miR-15b-5p | 4.02E-08 | 8.72E-08 | hsa-miR-345-5p | 2.53E-14 | 4.13E-13 | hsa-miR-769-5p | 2.59E-13 | 2.40E-12 |
| hsa-miR-16-5p | 0.0020 | 0.0027 | hsa-miR-346 | 0.0005 | 0.0008 | hsa-miR-874-3p | 5.84E-11 | 2.20E-10 |
| hsa-miR-17-5p | 0.0009 | 0.0012 | hsa-miR-34a-3p | 5.48E-09 | 1.41E-08 | hsa-miR-875-5p | 0.0029 | 0.0040 |
| hsa-miR-181a-2-3p | 4.56E-12 | 2.56E-11 | hsa-miR-34a-5p | 1.73E-16 | 1.49E-14 | hsa-miR-885-5p | 3.03E-09 | 8.38E-09 |
| hsa-miR-181a-3p | 3.21E-12 | 1.93E-11 | hsa-miR-34b-3p | 4.68E-12 | 2.59E-11 | hsa-miR-886-3p | 5.74E-11 | 2.19E-10 |
| hsa-miR-181a-5p | 8.36E-08 | 1.72E-07 | hsa-miR-361-3p | 6.53E-12 | 3.29E-11 | hsa-miR-886-5p | 5.51E-11 | 2.15E-10 |
| hsa-miR-181c-5p | 2.18E-05 | 3.54E-05 | hsa-miR-361-5p | 2.18E-08 | 4.93E-08 | hsa-miR-892b | 0.0014 | 0.0019 |
| hsa-miR-1825 | 0.0006 | 0.0009 | hsa-miR-362-5p | 7.42E-10 | 2.29E-09 | hsa-miR-92a-1-5p | 7.96E-10 | 2.44E-09 |
| hsa-miR-184 | 5.92E-05 | 9.31E-05 | hsa-miR-365a-3p | 1.34E-08 | 3.14E-08 | hsa-miR-92a-3p | 4.11E-15 | 1.17E-13 |
| hsa-miR-186-5p | 2.33E-06 | 4.03E-06 | hsa-miR-370-3p | 1.88E-11 | 8.48E-11 | hsa-miR-93-3p | 9.93E-12 | 4.94E-11 |
| hsa-miR-187-3p | 0.0008 | 0.0011 | hsa-miR-374a-5p | 6.96E-09 | 1.74E-08 | hsa-miR-93-5p | 9.44E-11 | 3.34E-10 |
| hsa-miR-18a-5p | 2.58E-07 | 4.86E-07 | hsa-miR-374b-5p | 2.23E-07 | 4.23E-07 | hsa-miR-939-5p | 1.18E-10 | 4.08E-10 |
| hsa-miR-190b | 3.44E-12 | 2.03E-11 | hsa-miR-375 | 6.35E-15 | 1.56E-13 | hsa-miR-9-3p | 0.0001 | 0.0002 |
| hsa-miR-191-3p | 1.38E-10 | 4.68E-10 | hsa-miR-376a-3p | 0.0067 | 0.0087 | hsa-miR-95-3p | 9.29E-05 | 0.0001 |
| hsa-miR-191-5p | 1.01E-07 | 2.04E-07 | hsa-miR-376a-5p | 0.0002 | 0.0003 | hsa-miR-98-5p | 2.99E-08 | 6.52E-08 |
| hsa-miR-193a-5p | 2.67E-06 | 4.60E-06 | hsa-miR-378a-3p | 0.0072 | 0.0093 | hsa-miR-99a-3p | 3.16E-06 | 5.36E-06 |
| hsa-miR-193b-3p | 6.98E-06 | 1.16E-05 | hsa-miR-380-3p | 3.46E-07 | 6.41E-07 | hsa-miR-99a-5p | 5.69E-05 | 9.00E-05 |
| hsa-miR-193b-5p | 0.0009 | 0.0012 | hsa-miR-381-3p | 2.55E-08 | 5.68E-08 | hsa-miR-99b-3p | 1.91E-06 | 3.32E-06 |
| hsa-miR-195-5p | 0.0032 | 0.0042 | hsa-miR-382-5p | 2.31E-13 | 2.20E-12 | hsa-miR-99b-5p | 2.09E-13 | 2.11E-12 |
| hsa-miR-197-3p | 3.80E-11 | 1.54E-10 | hsa-miR-409-3p | 1.03E-09 | 3.07E-09 |  |  |  |

Supplementary Table 6: 61 microRNAs were identified using the criteria: Up in TIF vs NIF (FDR<0.01) and expressed in more than 75% of serum samples

| MicroRNA | MicroRNA | MicroRNA | MicroRNA |
| --- | --- | --- | --- |
| hsa-let-7b-5p | hsa-miR-17-5p | hsa-miR-25-3p | hsa-miR-342-3p |
| hsa-let-7d-5p | hsa-miR-1825 | hsa-miR-26a-5p | hsa-miR-345-5p |
| hsa-let-7e-5p | hsa-miR-195-5p | hsa-miR-26b-5p | hsa-miR-374a-5p |
| hsa-miR-106b-5p | hsa-miR-197-3p | hsa-miR-27a-3p | hsa-miR-374b-5p |
| hsa-miR-126-3p | hsa-miR-199a-3p | hsa-miR-28-3p | hsa-miR-375 |
| hsa-miR-126-5p | hsa-miR-19a-3p | hsa-miR-29a-3p | hsa-miR-378a-3p |
| hsa-miR-1274B | hsa-miR-19b-3p | hsa-miR-301a-5p | hsa-miR-425-5p |
| hsa-miR-139-5p | hsa-miR-20b-5p | hsa-miR-30a-5p | hsa-miR-454-3p |
| hsa-miR-140-5p | hsa-miR-210-3p | hsa-miR-30b-5p | hsa-miR-484 |
| hsa-miR-142-3p | hsa-miR-21-5p | hsa-miR-30c-5p | hsa-miR-574-3p |
| hsa-miR-146a-5p | hsa-miR-221-3p | hsa-miR-30d-5p | hsa-miR-720 |
| hsa-miR-146b-5p | hsa-miR-222-3p | hsa-miR-30e-3p | hsa-miR-885-5p |
| hsa-miR-151a-3p | hsa-miR-223-3p | hsa-miR-320a | hsa-miR-892b |
| hsa-miR-151a-5P | hsa-miR-223-5p | hsa-miR-324-3p | hsa-miR-92a-3p |
| hsa-miR-16-5p | hsa-miR-24-3p | hsa-miR-331-3p | hsa-miR-93-5p |
|  |  |  | hsa-miR-99b-5p |

Supplementary Table 7: The difference in abundance of the 61 candidate microRNAs were tested using student’s t-test and 52 microRNAs showed significantly higher abundance in tumor mass vs TIF

| MicroRNA | t-test | FDR |  | MicroRNA | t-test | FDR |
| --- | --- | --- | --- | --- | --- | --- |
| hsa-miR-19a-3p | 3.04E-20 | 0.000172 |  | hsa-let-7b-5p | 6.58E-11 | 0.004655 |
| hsa-miR-451a | 3.26E-20 | 0.000345 |  | hsa-miR-342-3p | 4.69E-10 | 0.004828 |
| hsa-miR-126-3p | 2.94E-18 | 0.000517 |  | hsa-let-7d-5p | 6.15E-10 | 0.005 |
| hsa-miR-146b-5p | 9.34E-16 | 0.00069 |  | hsa-miR-151a-5P | 1.57E-09 | 0.005172 |
| hsa-miR-425-5p | 2.38E-15 | 0.000862 |  | hsa-miR-29a-3p | 2.08E-09 | 0.005345 |
| hsa-miR-21-5p | 7.54E-15 | 0.001034 |  | hsa-miR-30d-5p | 2.26E-08 | 0.005517 |
| hsa-miR-16-5p | 8.31E-15 | 0.001207 |  | hsa-miR-30a-5p | 4.05E-08 | 0.00569 |
| hsa-miR-25-3p | 1.87E-14 | 0.001379 |  | hsa-miR-140-5p | 2.08E-07 | 0.005862 |
| hsa-miR-223-3p | 2.32E-14 | 0.001552 |  | hsa-miR-92a-3p | 3.82E-07 | 0.006034 |
| hsa-miR-26a-5p | 3.01E-14 | 0.001724 |  | hsa-miR-374b-5p | 4.49E-07 | 0.006207 |
| hsa-miR-199a-5p | 4.58E-14 | 0.001897 |  | hsa-miR-20b-5p | 5.01E-07 | 0.006379 |
| hsa-miR-142-3p | 4.6E-14 | 0.002069 |  | hsa-miR-324-3p | 1.04E-06 | 0.006552 |
| hsa-miR-151a-3p | 1.35E-13 | 0.002241 |  | hsa-miR-320a | 1.81E-06 | 0.006724 |
| hsa-miR-26b-5p | 1.68E-13 | 0.002414 |  | hsa-miR-374a-5p | 2.23E-06 | 0.006897 |
| hsa-miR-30b-5p | 3.27E-13 | 0.002586 |  | hsa-let-7e-5p | 3.22E-06 | 0.007069 |
| hsa-miR-93-5p | 3.58E-13 | 0.002759 |  | hsa-miR-331-3p | 6.36E-06 | 0.007241 |
| hsa-miR-24-3p | 6.38E-13 | 0.002931 |  | hsa-miR-223-5p | 3.46E-05 | 0.007414 |
| hsa-miR-27a-3p | 8.69E-13 | 0.003103 |  | hsa-miR-301a-5p | 3.46E-05 | 0.007586 |
| hsa-miR-17-5p | 9.18E-13 | 0.003276 |  | hsa-miR-139-5p | 0.000118 | 0.007759 |
| hsa-miR-195-5p | 4.3E-12 | 0.003448 |  | hsa-miR-221-3p | 0.000163 | 0.007931 |
| hsa-miR-106b-5p | 7.8E-12 | 0.003621 |  | hsa-miR-885-5p | 0.000441 | 0.008103 |
| hsa-miR-99b-5p | 1.03E-11 | 0.003793 |  | hsa-miR-210-3p | 0.001922 | 0.008276 |
| hsa-miR-146a-5p | 4.34E-11 | 0.003966 |  | hsa-miR-454-3p | 0.002322 | 0.008448 |
| hsa-miR-20a-5p | 4.54E-11 | 0.004138 |  | hsa-miR-126-5p | 0.002912 | 0.008621 |
| hsa-miR-222-3p | 5.87E-11 | 0.00431 |  | hsa-miR-375 | 0.004257 | 0.008793 |
| hsa-miR-30c-5p | 6.32E-11 | 0.004483 |  | hsa-miR-30e-3p | 0.00623 | 0.008966 |

Supplementary Table 8: MicroRNA profiling in serum of Chinese breast cancer patients. The table show results from 52 of the 61 microRNA candidates identified in the TIF cohort. With FDR<0.05, 16 microRNAs were validated in the Chinese breast cancer cohort (marked with *). Nine microRNA were not at this platform, shown below the table (29).

| MicroRNA | BC/Healthy Volunteer | Direction in BC | P Value | P-value (Benjamini-Hochberg) |  | MicroRNA | BC/Healthy Volunteer | Direction in BC | P Value | P-value (Benjamini-Hochberg) |
| --- | --- | --- | --- | --- | --- | --- | --- | --- | --- | --- |
| hsa-miR-92a* | -1.34 | Up | 9.50E-08 | 8.97E-07 |  | **hsa-miR-28-3p** | 0.38 | Down | 0.064 | 0.123 |
| hsa-miR-223 | 0.98 | Down | 1.44E-06 | 1.80E-05 |  | **hsa-miR-30e** | -0.45 | Up | 0.093 | 0.149 |
| hsa-miR-16* | -1.30 | Up | 7.65E-06 | 2.68E-05 |  | **hsa-miR-126** | -0.20 | Up | 0.143 | 0.242 |
| hsa-miR-320a* | -0.98 | Up | 3.06E-05 | 0.0002 |  | **hsa-miR-21** | -0.33 | Up | 0.147 | 0.262 |
| hsa-miR-93* | -0.74 | Up | 0.0001 | 0.0005 |  | **hsa-miR-139-5p** | -0.31 | Up | 0.148 | 0.264 |
| hsa-miR-25* | -0.97 | Up | 0.0002 | 0.0007 |  | **hsa-miR-331-3p** | 0.22 | Down | 0.152 | 0.281 |
| hsa-miR-20b* | -1.37 | up | 0.0002 | 0.0011 |  | **hsa-miR-99b** | -0.27 | Up | 0.222 | 0.322 |
| hsa-miR-17* | -0.69 | Up | 0.0004 | 0.0011 |  | **hsa-miR-146b-5p** | 0.20 | Down | 0.273 | 0.413 |
| hsa-miR-142-3p | 0.66 | Down | 0.0008 | 0.0073 |  | **hsa-miR-27a** | 0.22 | Down | 0.292 | 0.439 |
| hsa-miR-374b | 0.42 | Down | 0.0019 | 0.0073 |  | **hsa-miR-324-3p** | -0.20 | Up | 0.336 | 0.484 |
| hsa-miR-195* | -0.94 | Up | 0.0023 | 0.0073 |  | **hsa-miR-140-5p** | 0.18 | Up | 0.372 | 0.520 |
| hsa-miR-199a-5p | 0.74 | Down | 0.0026 | 0.0127 |  | **hsa-let-7b-5p** | -0.23 | Up | 0.415 | 0.541 |
| hsa-miR-378* | -0.95 | Up | 0.0032 | 0.0128 |  | **hsa-miR-29a** | 0.20 | Down | 0.470 | 0.592 |
| hsa-miR-30d* | -0.54 | Up | 0.0035 | 0.0084 |  | **hsa-miR-301a** | 0.12 | Down | 0.501 | 0.605 |
| hsa-miR-484* | -0.50 | Up | 0.0037 | 0.0078 |  | **hsa-miR-26b** | -0.10 | Up | 0.504 | 0.596 |
| hsa-miR-574-3p* | -0.60 | Up | 0.0053 | 0.0269 |  | **hsa-miR-885-5p** | 0.36 | Down | 0.505 | 0.619 |
| hsa-miR-30a* | -0.84 | Up | 0.0064 | 0.0285 |  | **hsa-miR-26a** | 0.12 | Down | 0.536 | 0.624 |
| hsa-miR-19a* | -0.80 | Up | 0.0065 | 0.0163 |  | **hsa-miR-151-5p** | 0.07 | Down | 0.558 | 0.669 |
| hsa-miR-30b | 0.33 | Down | 0.0081 | 0.0271 |  | **hsa-miR-720** | -0.15 | Up | 0.562 | 0.695 |
| hsa-miR-425* | -0.51 | Up | 0.0089 | 0.0199 |  | **hsa-miR-106b** | -0.14 | Up | 0.577 | 0.669 |
| hsa-miR-210* | -0.72 | Up | 0.0161 | 0.0443 |  | **hsa-miR-221** | 0.10 | Down | 0.582 | 0.664 |
| hsa-miR-223 | 0.58 | Down | 0.0349 | 0.0731 |  | **hsa-miR-342-3p** | 0.09 | Down | 0.631 | 0.721 |
| hsa-miR-197 | 0.35 | Down | 0.0465 | 0.0816 |  | **hsa-miR-30c** | 0.02 | Down | 0.854 | 0.902 |
| hsa-miR-24 | -0.30 | Up | 0.0546 | 0.1185 |  | **hsa-let-7e** | 0.05 | Down | 0.856 | 0.902 |
| hsa-let-7d | -0.20 | Up | 0.0578 | 0.1512 |  | **hsa-miR-146a** | -0.03 | Up | 0.882 | 0.918 |
| hsa-miR-222 | -0.38 | Up | 0.0623 | 0.1354 |  | **hsa-miR-375** | 0.03 | Down | 0.954 | 0.969 |

Not at the platform:

| hsa-miR-126-5p |
| --- |
| hsa-miR-1274B |
| hsa-miR-151a-3p |
| hsa-miR-345-5p |
| hsa-miR-374a-5p |
| hsa-miR-454-3p |
| hsa-miR-892b |
| hsa-miR-1825 |
| hsa-miR-19b-3p |

Supplementary Table 9: Out of the 457 microRNA in TIF, the presence of TILs and tumor percentage contributed significantly to the variation of 179 microRNAs (FDR<0.05)

| MicroRNA | p | adj_p | r | adj_r |  | MicroRNA | p | adj_p | r | adj_r |  | MicroRNA | p | adj_p | r | adj_r |
| --- | --- | --- | --- | --- | --- | --- | --- | --- | --- | --- | --- | --- | --- | --- | --- | --- |
| hsa-miR-125a-5p | 0.00 | 0.00 | 0.55 | 0.53 |  | **hsa-miR-340-5p** | 0.00 | 0.00 | 0.22 | 0.19 |  | **hsa-miR-629-3p** | 0.01 | 0.01 | 0.16 | 0.13 |
| hsa-miR-769-5p | 0.00 | 0.00 | 0.54 | 0.52 |  | **hsa-miR-148a-3p** | 0.00 | 0.00 | 0.22 | 0.19 |  | **hsa-miR-375** | 0.01 | 0.01 | 0.16 | 0.13 |
| hsa-miR-491-5p | 0.00 | 0.00 | 0.49 | 0.48 |  | **hsa-miR-374a-5p** | 0.00 | 0.00 | 0.22 | 0.19 |  | **hsa-miR-590-3P** | 0.01 | 0.01 | 0.16 | 0.13 |
| hsa-miR-324-5p | 0.00 | 0.00 | 0.46 | 0.44 |  | **hsa-miR-744-5p** | 0.00 | 0.00 | 0.22 | 0.19 |  | **hsa-miR-15b-5p** | 0.01 | 0.01 | 0.16 | 0.13 |
| hsa-miR-1300 | 0.00 | 0.00 | 0.46 | 0.44 |  | **hsa-miR-500a-5p** | 0.00 | 0.00 | 0.22 | 0.19 |  | **hsa-miR-30b-5p** | 0.01 | 0.01 | 0.16 | 0.13 |
| hsa-miR-296-5p | 0.00 | 0.00 | 0.43 | 0.41 |  | **hsa-miR-151a-5P** | 0.00 | 0.00 | 0.21 | 0.19 |  | **hsa-miR-495-3p** | 0.01 | 0.01 | 0.16 | 0.13 |
| hsa-miR-423-5p | 0.00 | 0.00 | 0.40 | 0.38 |  | **hsa-miR-200b-3p** | 0.00 | 0.00 | 0.21 | 0.19 |  | **hsa-miR-10a-5p** | 0.01 | 0.01 | 0.16 | 0.13 |
| hsa-miR-190b | 0.00 | 0.00 | 0.40 | 0.37 |  | **hsa-miR-141-3p** | 0.00 | 0.00 | 0.21 | 0.18 |  | **hsa-miR-126-5p** | 0.01 | 0.01 | 0.16 | 0.13 |
| hsa-miR-142-3p | 0.00 | 0.00 | 0.39 | 0.37 |  | **hsa-miR-301a-5p** | 0.00 | 0.01 | 0.21 | 0.18 |  | **hsa-miR-130b-3p** | 0.01 | 0.02 | 0.16 | 0.13 |
| hsa-miR-210-3p | 0.00 | 0.00 | 0.39 | 0.37 |  | **hsa-miR-95-3p** | 0.00 | 0.01 | 0.21 | 0.18 |  | **hsa-miR-424-5p** | 0.01 | 0.02 | 0.16 | 0.13 |
| hsa-miR-155-5p | 0.00 | 0.00 | 0.38 | 0.36 |  | **hsa-miR-484** | 0.00 | 0.01 | 0.21 | 0.18 |  | **hsa-miR-518e-3p** | 0.01 | 0.02 | 0.16 | 0.13 |
| hsa-miR-1183 | 0.00 | 0.00 | 0.38 | 0.36 |  | **hsa-miR-330-5p** | 0.00 | 0.01 | 0.21 | 0.18 |  | **hsa-miR-99b-3p** | 0.01 | 0.02 | 0.16 | 0.13 |
| hsa-miR-324-3p | 0.00 | 0.00 | 0.36 | 0.34 |  | **hsa-miR-1271-5p** | 0.00 | 0.01 | 0.21 | 0.18 |  | **hsa-miR-370-3p** | 0.01 | 0.02 | 0.16 | 0.13 |
| hsa-miR-1276 | 0.00 | 0.00 | 0.36 | 0.33 |  | **hsa-miR-33a-3p** | 0.00 | 0.01 | 0.20 | 0.18 |  | **hsa-miR-93-3p** | 0.01 | 0.02 | 0.16 | 0.13 |
| hsa-miR-34a-5p | 0.00 | 0.00 | 0.35 | 0.33 |  | **hsa-miR-376a-5p** | 0.00 | 0.01 | 0.20 | 0.18 |  | **hsa-miR-223-5p** | 0.01 | 0.02 | 0.16 | 0.13 |
| hsa-miR-151a-3p | 0.00 | 0.00 | 0.35 | 0.32 |  | **hsa-miR-340-3p** | 0.00 | 0.01 | 0.20 | 0.17 |  | **hsa-miR-142-5p** | 0.01 | 0.02 | 0.16 | 0.13 |
| hsa-miR-505-5p | 0.00 | 0.00 | 0.34 | 0.32 |  | **hsa-miR-342-3p** | 0.00 | 0.01 | 0.20 | 0.17 |  | **hsa-miR-214-5p** | 0.01 | 0.02 | 0.16 | 0.12 |
| hsa-miR-345-5p | 0.00 | 0.00 | 0.34 | 0.32 |  | **hsa-miR-181a-2-3p** | 0.00 | 0.01 | 0.20 | 0.17 |  | **hsa-miR-326** | 0.01 | 0.02 | 0.15 | 0.12 |
| hsa-miR-145-5p | 0.00 | 0.00 | 0.34 | 0.32 |  | **hsa-miR-362-5p** | 0.00 | 0.01 | 0.20 | 0.17 |  | **hsa-miR-132-3p** | 0.01 | 0.02 | 0.15 | 0.12 |
| hsa-miR-28-3p | 0.00 | 0.00 | 0.34 | 0.32 |  | **hsa-miR-590-5p** | 0.00 | 0.01 | 0.20 | 0.17 |  | **hsa-miR-22-5p** | 0.01 | 0.02 | 0.15 | 0.12 |
| hsa-miR-1180-3p | 0.00 | 0.00 | 0.33 | 0.31 |  | **hsa-miR-221-3p** | 0.00 | 0.01 | 0.19 | 0.17 |  | **hsa-let-7d-5p** | 0.01 | 0.02 | 0.15 | 0.12 |
| hsa-miR-21-3p | 0.00 | 0.00 | 0.32 | 0.30 |  | **hsa-miR-23a-3p** | 0.00 | 0.01 | 0.19 | 0.17 |  | **hsa-miR-596** | 0.01 | 0.02 | 0.15 | 0.12 |
| hsa-miR-146b-3p | 0.00 | 0.00 | 0.31 | 0.29 |  | **hsa-miR-487b-3p** | 0.00 | 0.01 | 0.19 | 0.16 |  | **hsa-miR-100-5p** | 0.01 | 0.02 | 0.15 | 0.12 |
| hsa-miR-30d-5p | 0.00 | 0.00 | 0.30 | 0.28 |  | **hsa-miR-222-3p** | 0.00 | 0.01 | 0.19 | 0.16 |  | **hsa-miR-143-3p** | 0.01 | 0.02 | 0.15 | 0.12 |
| hsa-miR-30d-5p-1 | 0.00 | 0.00 | 0.30 | 0.28 |  | **hsa-miR-200a-3p** | 0.00 | 0.01 | 0.19 | 0.16 |  | **hsa-miR-7-5p** | 0.01 | 0.02 | 0.15 | 0.12 |
| hsa-miR-622 | 0.00 | 0.00 | 0.30 | 0.27 |  | **hsa-miR-30a-3p** | 0.00 | 0.01 | 0.19 | 0.16 |  | **hsa-miR-19a-3p** | 0.01 | 0.02 | 0.15 | 0.12 |
| hsa-miR-224-5p | 0.00 | 0.00 | 0.27 | 0.25 |  | **hsa-miR-205-5p** | 0.00 | 0.01 | 0.19 | 0.16 |  | **hsa-miR-18a-5p** | 0.01 | 0.02 | 0.15 | 0.12 |
| hsa-miR-874-3p | 0.00 | 0.00 | 0.27 | 0.25 |  | **hsa-miR-29b-3p** | 0.00 | 0.01 | 0.19 | 0.16 |  | **hsa-miR-1282** | 0.01 | 0.02 | 0.15 | 0.12 |
| hsa-miR-409-3p | 0.00 | 0.00 | 0.27 | 0.24 |  | **hsa-miR-191-3p** | 0.00 | 0.01 | 0.19 | 0.16 |  | **hsa-miR-30a-5p** | 0.01 | 0.02 | 0.15 | 0.11 |
| hsa-let-7b-5p | 0.00 | 0.00 | 0.27 | 0.24 |  | **hsa-miR-625-5p** | 0.00 | 0.01 | 0.19 | 0.16 |  | **hsa-miR-26b-5p** | 0.01 | 0.02 | 0.15 | 0.11 |
| hsa-miR-361-3p | 0.00 | 0.00 | 0.27 | 0.24 |  | **hsa-miR-708-5p** | 0.00 | 0.01 | 0.19 | 0.16 |  | **hsa-miR-7-1-3p** | 0.01 | 0.02 | 0.15 | 0.11 |
| hsa-miR-181a-5p | 0.00 | 0.00 | 0.27 | 0.24 |  | **hsa-miR-365a-3p** | 0.00 | 0.01 | 0.19 | 0.16 |  | **hsa-miR-361-5p** | 0.01 | 0.02 | 0.14 | 0.11 |
| hsa-miR-548d-5p | 0.00 | 0.00 | 0.27 | 0.24 |  | **hsa-miR-17-5p** | 0.00 | 0.01 | 0.19 | 0.16 |  | **hsa-miR-140-3p** | 0.01 | 0.02 | 0.14 | 0.11 |
| hsa-miR-494-3p | 0.00 | 0.00 | 0.27 | 0.24 |  | **hsa-miR-424-3p** | 0.00 | 0.01 | 0.19 | 0.16 |  | **hsa-miR-382-5p** | 0.01 | 0.02 | 0.14 | 0.11 |
| hsa-miR-146b-5p | 0.00 | 0.00 | 0.26 | 0.24 |  | **hsa-miR-27a-5p** | 0.00 | 0.01 | 0.19 | 0.16 |  | **hsa-miR-126-3p** | 0.01 | 0.02 | 0.14 | 0.11 |
| hsa-miR-1275 | 0.00 | 0.00 | 0.26 | 0.24 |  | **hsa-miR-455-5p** | 0.00 | 0.01 | 0.19 | 0.16 |  | **hsa-miR-27b-3p** | 0.01 | 0.02 | 0.14 | 0.11 |
| hsa-miR-200c-3p | 0.00 | 0.00 | 0.26 | 0.24 |  | **hsa-miR-28-5p** | 0.00 | 0.01 | 0.18 | 0.16 |  | **hsa-miR-26a-5p** | 0.02 | 0.03 | 0.14 | 0.11 |
| hsa-miR-331-5p | 0.00 | 0.00 | 0.26 | 0.23 |  | **hsa-miR-320a** | 0.00 | 0.01 | 0.18 | 0.16 |  | **hsa-let-7e-5p** | 0.02 | 0.03 | 0.14 | 0.11 |
| hsa-miR-193b-5p | 0.00 | 0.00 | 0.25 | 0.23 |  | **hsa-miR-125b-5p** | 0.00 | 0.01 | 0.18 | 0.15 |  | **hsa-miR-23a-5p** | 0.02 | 0.03 | 0.14 | 0.11 |
| hsa-miR-128-3p | 0.00 | 0.00 | 0.25 | 0.23 |  | **hsa-let-7a-5p** | 0.00 | 0.01 | 0.18 | 0.15 |  | **hsa-miR-98-5p** | 0.02 | 0.03 | 0.13 | 0.10 |
| hsa-miR-339-3p | 0.00 | 0.00 | 0.25 | 0.22 |  | **hsa-miR-106b-5p** | 0.00 | 0.01 | 0.18 | 0.15 |  | **hsa-miR-643** | 0.02 | 0.03 | 0.13 | 0.10 |
| hsa-miR-320B | 0.00 | 0.00 | 0.25 | 0.22 |  | **hsa-miR-532-3p** | 0.00 | 0.01 | 0.18 | 0.15 |  | **hsa-miR-130a-3p** | 0.02 | 0.03 | 0.13 | 0.10 |
| hsa-miR-576-3p | 0.00 | 0.00 | 0.25 | 0.22 |  | **hsa-miR-30d-3p** | 0.00 | 0.01 | 0.18 | 0.15 |  | **hsa-miR-1244** | 0.02 | 0.03 | 0.13 | 0.10 |
| hsa-miR-660-5p | 0.00 | 0.00 | 0.25 | 0.22 |  | **hsa-miR-564** | 0.00 | 0.01 | 0.18 | 0.15 |  | **hsa-miR-639** | 0.02 | 0.03 | 0.13 | 0.10 |
| hsa-miR-532-5p | 0.00 | 0.00 | 0.24 | 0.22 |  | **hsa-miR-181a-3p** | 0.00 | 0.01 | 0.18 | 0.15 |  | **hsa-miR-92a-1-5p** | 0.02 | 0.03 | 0.13 | 0.10 |
| hsa-miR-27a-3p | 0.00 | 0.00 | 0.24 | 0.22 |  | **hsa-miR-197-3p** | 0.00 | 0.01 | 0.18 | 0.15 |  | **hsa-miR-16-5p** | 0.02 | 0.04 | 0.13 | 0.10 |
| hsa-miR-335-3p | 0.00 | 0.00 | 0.24 | 0.22 |  | **hsa-miR-20b-5p** | 0.00 | 0.01 | 0.18 | 0.15 |  | **hsa-miR-206** | 0.02 | 0.04 | 0.13 | 0.10 |
| hsa-miR-19b-3p | 0.00 | 0.00 | 0.24 | 0.21 |  | **hsa-miR-642a-5p** | 0.00 | 0.01 | 0.18 | 0.15 |  | **hsa-miR-429** | 0.02 | 0.04 | 0.13 | 0.09 |
| hsa-miR-454-3p | 0.00 | 0.00 | 0.24 | 0.21 |  | **hsa-miR-106b-3p** | 0.00 | 0.01 | 0.17 | 0.14 |  | **hsa-miR-548b-5p** | 0.02 | 0.04 | 0.12 | 0.09 |
| hsa-miR-29a-3p | 0.00 | 0.00 | 0.23 | 0.21 |  | **hsa-miR-223-3p** | 0.01 | 0.01 | 0.17 | 0.14 |  | **hsa-let-7f-5p** | 0.02 | 0.04 | 0.12 | 0.09 |
| hsa-miR-127-3p | 0.00 | 0.00 | 0.23 | 0.20 |  | **hsa-miR-574-3p** | 0.01 | 0.01 | 0.17 | 0.14 |  | **hsa-miR-106a-5p** | 0.02 | 0.04 | 0.12 | 0.09 |
| hsa-miR-25-3p | 0.00 | 0.00 | 0.23 | 0.20 |  | **hsa-miR-203a-3p** | 0.01 | 0.01 | 0.17 | 0.14 |  | **hsa-miR-212-3p** | 0.02 | 0.04 | 0.12 | 0.09 |
| hsa-let-7g-5p | 0.00 | 0.00 | 0.23 | 0.20 |  | **hsa-miR-30e-3p** | 0.01 | 0.01 | 0.17 | 0.14 |  | **hsa-miR-139-5p** | 0.03 | 0.04 | 0.12 | 0.09 |
| hsa-miR-31-5p | 0.00 | 0.00 | 0.23 | 0.20 |  | **hsa-miR-125a-3p** | 0.01 | 0.01 | 0.17 | 0.14 |  | **hsa-miR-339-5p** | 0.03 | 0.04 | 0.12 | 0.09 |
| hsa-miR-93-5p | 0.00 | 0.00 | 0.23 | 0.20 |  | **hsa-miR-10b-3p** | 0.01 | 0.01 | 0.17 | 0.14 |  | **hsa-miR-1254** | 0.03 | 0.04 | 0.12 | 0.09 |
| hsa-miR-214-3p | 0.00 | 0.00 | 0.23 | 0.20 |  | **hsa-miR-652-3p** | 0.01 | 0.01 | 0.17 | 0.14 |  | **hsa-miR-501-5p** | 0.03 | 0.04 | 0.12 | 0.09 |
| hsa-miR-24-3p | 0.00 | 0.00 | 0.22 | 0.20 |  | **hsa-miR-1285-3p** | 0.01 | 0.01 | 0.17 | 0.14 |  | **hsa-miR-769-3p** | 0.03 | 0.05 | 0.11 | 0.08 |
| hsa-miR-939-5p | 0.00 | 0.00 | 0.22 | 0.20 |  | **hsa-miR-744-3p** | 0.01 | 0.01 | 0.16 | 0.13 |  | **hsa-miR-31-3p** | 0.03 | 0.05 | 0.11 | 0.08 |
| hsa-miR-331-3p | 0.00 | 0.00 | 0.22 | 0.19 |  | **hsa-miR-10b-5p** | 0.01 | 0.01 | 0.16 | 0.13 |  | **hsa-miR-425-5p** | 0.04 | 0.05 | 0.11 | 0.08 |
|  |  |  |  |  |  |  |  |  |  |  |  | **hsa-miR-99a-3p** | 0.04 | 0.05 | 0.11 | 0.08 |
|  |  |  |  |  |  |  |  |  |  |  |  | **hsa-miR-1274A** | 0.04 | 0.05 | 0.11 | 0.08 |

Supplementary Table 10: Pathway analyses were performed for microRNAs significantly correlated with subgroups of TILs and adipocytes. Top Canonical pathways included p-value are shown for each analysis. Number of microRNAs included in the analysis are shown in brackets.

| **Top 5 Canonical Pathways** |  |
| --- | --- |
|  |  |
| **TIL corr TIF (16miR)** | **p-value** |
| Molecular Mechanisms of Cancer | 3.41E-28 |
| Role of Macrophages Fibroblasts and Endothelial Cells in Rheumatoid Arthritis | 1.54E-26 |
| Colorectal Cancer Metastasis Signaling | 1.62E-24 |
| Pancreatic Adenocarcinoma Signaling | 1.76E-22 |
| Chronic Myeloid Leukemia Signaling | 2.40E-20 |
|  |  |
| **CD4+ corr TIF (6miR)** | **p-value** |
| Hepatic Fibrosis / Hepatic Stellate Cell Activation | 1.11E-25 |
| Role of Macrophages Fibroblasts and Endothelial Cells in Rheumatoid Arthritis | 7.02E-24 |
| Dendritic Cell Maturation | 1.97E-23 |
| IL-6 Signaling | 6.75E-22 |
| IL-10 Signaling | 1.27E-19 |
|  |  |
| **CD8+ corr TIF (2miR)** | **p-value** |
| Molecular Mechanisms of Cancer | 2.59E-10 |
| Glucocorticoid Receptor Signaling | 8.32E-10 |
| PTEN Signaling | 4.94E-09 |
| PI3K/AKT Signaling | 7.29E-09 |
| IL-8 Signaling | 7.25E-08 |
|  |  |
| **CD45+ corr TIF (2miR)** | **p-value** |
| Molecular Mechanisms of Cancer | 2.43E-06 |
| PPAR/RXR Activation | 4.38E-06 |
| Cardiac Hypertrophy Signaling | 1.12E-05 |
| Phospholipase C Signaling | 1.39E-05 |
| Cholecystokinin/Gastrin-mediated Signaling | 3.83E-05 |
|  |  |
| **CD3+ corr TIF (4miR)** | **p-value** |
| Molecular Mechanisms of Cancer | 2.15E-17 |
| Pancreatic Adenocarcinoma Signaling | 3.92E-17 |
| Estrogen-mediated S-phase Entry | 1.39E-13 |
| Chronic Myeloid Leukemia Signaling | 1.60E-12 |
| Glioma Signaling | 5.32E-12 |
|  |  |
| **CD68+ corr TIF (6miR)** | **p-value** |
| Molecular Mechanisms of Cancer | 3.03E-24 |
| Pancreatic Adenocarcinoma Signaling | 2.68E-21 |
| Chronic Myeloid Leukemia Signaling | 1.89E-15 |
| Cell Cycle: G1/S Checkpoint Regulation | 6.30E-14 |
| p53 Signaling | 1.46E-13 |
|  |  |
| **Adiposcytes corr TIF (7miR)** | **p-value** |
| Molecular Mechanisms of Cancer | 4.50E-17 |
| p53 Signaling | 9.36E-15 |
| Aryl Hydrocarbon Receptor Signaling | 1.60E-14 |
| Estrogen-mediated S-phase Entry | 3.32E-13 |
| Pancreatic Adenocarcinoma Signaling | 7.97E-13 |

Supplementary Table 11: Kruskal-Wallis Anova test was performed to identify microRNAs with differential distribution between the subgroups. P-value < 0.05 is considered significant.

| **Her2 - LumA** | **p-value** |  | **Her2 - lumB** | **p-value** | |  | **Her2 - TNBC** | **p-value** |
| --- | --- | --- | --- | --- | --- | --- | --- | --- |
| hsa-miR-671-3p | 0.001 |  | hsa-miR-671-3p | | 0.01 |  | hsa-miR-671-3p | 0.001 |
| hsa-miR-30a-3p | 0.001 |  | hsa-miR-432-3p | | 0.01 |  | hsa-miR-432-3p | 0.01 |
| hsa-miR-576-3p | 0.001 |  | hsa-miR-524-3p | | 0.01 |  | hsa-miR-589-5p | 0.01 |
| hsa-miR-30e-3p | 0.001 |  | hsa-miR-125b-1-3p | | 0.02 |  | hsa-miR-549a | 0.02 |
| hsa-miR-224-5p | 0.01 |  | hsa-miR-576-3p | | 0.02 |  | hsa-miR-331-5p | 0.02 |
| hsa-miR-299-5p | 0.01 |  | hsa-miR-330-5p | | 0.02 |  | hsa-miR-622 | 0.02 |
| hsa-miR-628-5p | 0.01 |  | hsa-miR-361-5p | | 0.02 |  | hsa-miR-342-5p | 0.03 |
| hsa-miR-376a-5p | 0.01 |  | hsa-miR-646 | | 0.02 |  | hsa-miR-339-3p | 0.03 |
| hsa-miR-1282 | 0.02 |  | hsa-miR-190b | | 0.02 |  | hsa-miR-184 | 0.04 |
| hsa-miR-342-3p | 0.02 |  | hsa-miR-342-3p | | 0.02 |  | hsa-miR-375 | 0.04 |
| hsa-miR-625-3p | 0.02 |  | hsa-miR-342-5p | | 0.03 |  | hsa-miR-455-3p | 0.05 |
| hsa-miR-369-3p | 0.02 |  | hsa-miR-99a-5p | | 0.03 |  | hsa-miR-17-3p | 0.05 |
| hsa-miR-570-3p | 0.02 |  | hsa-miR-1179 | | 0.04 |  | hsa-miR-125a-3p | 0.05 |
| hsa-miR-1179 | 0.02 |  | hsa-miR-27a-5p | | 0.04 |  | hsa-miR-338-3p | 0.05 |
| hsa-miR-505-5p | 0.03 |  | hsa-miR-224-5p | | 0.04 |  |  |  |
| hsa-miR-1274A | 0.03 |  | hsa-miR-494-3p | | 0.04 |  | **LumA - TNBC** | **p-value** |
| hsa-miR-500a-5p | 0.03 |  | hsa-miR-885-3p | | 0.04 |  | hsa-miR-376a-5p | 0.001 |
| hsa-miR-519e-3p | 0.03 |  | hsa-miR-376a-5p | | 0.04 |  | hsa-miR-190b | 0.001 |
| hsa-miR-362-5p | 0.03 |  | hsa-miR-1825 | | 0.05 |  | hsa-miR-375 | 0.001 |
| hsa-miR-30d-3p | 0.03 |  | hsa-miR-589-5p | | 0.05 |  | hsa-miR-432-3p | 0.001 |
| hsa-miR-342-5p | 0.03 |  |  | |  |  | hsa-miR-423-5p | 0.001 |
| hsa-miR-190b | 0.03 |  |  | |  |  | hsa-miR-224-5p | 0.001 |
| hsa-miR-148a-5p | 0.03 |  | **LumA - LumB** | | **p-value** |  | hsa-miR-625-3p | 0.001 |
| hsa-miR-191-3p | 0.03 |  | hsa-miR-432-3p | | 0.01 |  | hsa-miR-519a | 0.01 |
| hsa-miR-485-3p | 0.04 |  | hsa-miR-485-3p | | 0.01 |  | hsa-miR-452-5p | 0.01 |
| hsa-miR-425-5p | 0.05 |  | hsa-miR-1282 | | 0.01 |  | hsa-miR-874-3p | 0.01 |
| hsa-miR-23a-5p | 0.05 |  | hsa-miR-519e-3p | | 0.01 |  | hsa-miR-142-3p | 0.01 |
| hsa-miR-130b-3p | 0.05 |  | hsa-let-7b-5p | | 0.01 |  | hsa-miR-1282 | 0.01 |
|  |  |  | hsa-miR-591 | | 0.02 |  | hsa-miR-184 | 0.01 |
| **LumB - TNBC** | **p-value** |  | hsa-miR-539-5p | | 0.02 |  | hsa-miR-505-5p | 0.01 |
| hsa-miR-190b | 0.001 |  | hsa-miR-524-3p | | 0.02 |  | hsa-miR-378a-3p | 0.01 |
| hsa-miR-452-5p | 0.01 |  | hsa-miR-30d-3p | | 0.03 |  | hsa-miR-133a-3p | 0.01 |
| hsa-miR-146a-5p | 0.01 |  | hsa-miR-765 | | 0.03 |  | hsa-miR-146b-3p | 0.02 |
| hsa-miR-376a-5p | 0.01 |  | hsa-miR-516a-3p | | 0.03 |  | hsa-miR-9-5p | 0.02 |
| hsa-miR-524-3p | 0.01 |  | hsa-miR-483-5p | | 0.03 |  | hsa-let-7b-5p | 0.02 |
| hsa-miR-885-3p | 0.01 |  | hsa-miR-483-3p | | 0.03 |  | hsa-miR-519b-3p | 0.02 |
| hsa-miR-224-5p | 0.01 |  | hsa-miR-217 | | 0.04 |  | hsa-miR-335-5p | 0.02 |
| hsa-miR-361-5p | 0.01 |  | hsa-miR-370-3p | | 0.04 |  | hsa-miR-155-5p | 0.02 |
| hsa-miR-519a | 0.02 |  | hsa-miR-323a-3p | | 0.04 |  | hsa-miR-196b-5p | 0.03 |
| hsa-miR-9-5p | 0.02 |  | hsa-miR-1227-3p | | 0.04 |  | hsa-miR-575 | 0.03 |
| hsa-miR-378a-3p | 0.02 |  | hsa-miR-27a-5p | | 0.05 |  | hsa-miR-138-5p | 0.03 |
| hsa-miR-153-3p | 0.02 |  | hsa-miR-133a-3p | | 0.05 |  | hsa-miR-17-5p | 0.03 |
| hsa-miR-146b-3p | 0.03 |  | hsa-miR-939-5p | | 0.05 |  | hsa-miR-135b-5p | 0.03 |
| hsa-miR-342-3p | 0.03 |  |  | |  |  | hsa-miR-342-3p | 0.04 |
| hsa-miR-155-5p | 0.04 |  |  | |  |  | hsa-miR-1296-5p | 0.04 |
| hsa-miR-135b-5p | 0.04 |  |  | |  |  | hsa-miR-638 | 0.04 |
| hsa-miR-335-5p | 0.05 |  |  | |  |  | hsa-miR-422a | 0.05 |
|  |  |  |  | |  |  | hsa-miR-885-5p | 0.05 |
|  |  |  |  | |  |  | hsa-miR-296-5p | 0.05 |
|  |  |  |  | |  |  | hsa-miR-146b-5p | 0.05 |
|  |  |  |  | |  |  | hsa-miR-548j-5p | 0.05 |
|  |  |  |  | |  |  | hsa-miR-130b-5p | 0.05 |
|  |  |  |  | |  |  | hsa-miR-18a-3p | 0.05 |
|  |  |  |  | |  |  | hsa-miR-516a-3p | 0.05 |
|  |  |  |  | |  |  | hsa-miR-20b-5p | 0.05 |
|  |  |  |  | |  |  | hsa-miR-30a-3p | 0.05 |
|  |  |  |  | |  |  |  |  |
